# Delivery of exogenous mitochondria via centrifugation enhances cellular metabolic function

**DOI:** 10.1038/s41598-018-21539-y

**Published:** 2018-02-20

**Authors:** Mi Jin Kim, Jung Wook Hwang, Chang-Koo Yun, Youngjun Lee, Yong-Soo Choi

**Affiliations:** 1 Department of Biotechnology, CHA University, Seongnam, 13488 Republic of Korea; 2 Paean Biotechnology Inc, Daejeon, 34028 Republic of Korea

## Abstract

Mitochondria are essential organelles involved in the maintenance of cell growth and function, and have been investigated as therapeutic targets in various diseases. Recent studies have demonstrated that direct mitochondrial transfer can restore cellular functions of cells with inherited or acquired mitochondrial dysfunction. However, previous mitochondrial transfer methods are inefficient and time-consuming. Here, we developed a simple and easy mitochondrial transfer protocol using centrifugation, which can be applied to any cell type. By our simple centrifugation method, we found that the isolated mitochondria could be successfully transferred into target cells, including mitochondrial DNA-deleted Rho^0^ cells and dexamethasone-treated atrophic muscle cells. We found that mitochondrial transfer normalised ATP production, mitochondrial membrane potential, mitochondrial reactive oxygen species level, and the oxygen consumption rate of the target cells. Furthermore, delivery of intact mitochondria blocked the AMPK/FoxO3/Atrogene pathway underlying muscle atrophy in atrophic muscle cells. Taken together, this simple and rapid mitochondrial transfer method can be used to treat mitochondrial dysfunction-related diseases.

## Introduction

Mitochondria are powerful and dynamic organelles responsible for essential cell functions, including energy metabolism, generation of free radicals, maintenance of calcium homeostasis, cell survival and death. Mitochondrial dysfunction is being recognized as being involved with many serious health problems such as aging^1^, cancer^2^, metabolic disorders^3^ and neurodegenerative diseases^4^. Muscle disorders such as muscle atrophy, degeneration and myopathy are also caused by mitochondrial malfunction^5,6^. Abnormal activities of enzymes of the mitochondrial respiratory chain and mitochondrial DNA (mtDNA) deletions have been observed in aged skeletal muscles^7^. These mtDNA mutations cause cellular dysfunction and lead to loss of muscle mass and strength. Oxidative damage resulting from errors in mtDNA replication and the repair system are thought to be at the root cause of these diseases^8^. Although mitochondrial dysfunction and muscle disorders are closely related, the detailed underlying mechanisms remain enigmatic.

Diverse mechanisms lead to mitochondrial dysfunction, including changes in the nuclear or mitochondrial genome, environmental insults or alterations in homeostasis^9^. Accumulation of dysfunctional mitochondria (>70–80%) upon exposure to intracellular or extracellular stress leads to oxidative stress, and in turn, affects intracellular signalling and gene expression^6,10^. Under severe oxidative stress, ATP is depleted, which prevents controlled apoptotic death and instead causes necrosis^11^. A recent study indicates that increased production of mitochondrial reactive oxygen species (mROS) is a major contributor to mitochondrial damage and dysfunction associated with prolonged skeletal muscle inactivity^6^. In addition, increased mitochondrial fragmentation caused by mROS production results in cellular energy stress (e.g., a low ATP level) and activation of the AMPK-FoxO3 signalling pathway, which induces expression of atrophy-related genes, protein breakdown and ultimately muscle atrophy^5,6,12^. Collectively, these results indicate that modulation of mROS production plays a major role in the prevention of muscle atrophy. Although recent studies provide direct evidence linking mitochondrial signalling with muscle atrophy, no mitochondria-targeted therapy to ameliorate muscle atrophy has been developed to date.

Existing mitochondria-targeted therapeutic strategies can be categorised as follows: 1) repair via scavenging of mROS, 2) reprogramming via stimulation of the mitochondrial regulatory program and 3) replacement via transfer of healthy exogenous mitochondria^13^. However, since modulation of mitochondrial function via repair and reprogramming can’t overcome genetic defects, replacement of damaged mitochondria represents an attractive option^14^. In this regard, recent studies have shown that the healthy or modified mitochondria can be delivered to damaged cells, restoring cellular function and treating the disease^15–20^. There have also been reports of direct delivery of healthy mitochondria to specific cells *in vitro*^21–23^. However, these methods have limitations in terms of efficiency and require cell cultivation process for mitochondrial delivery.

In this study, we developed a simple method to transfer mitochondria into cells by first mixing them together followed by centrifugation. This method makes mitochondrial delivery possible into any cell type, and no additional incubation is required. The transfer efficiency remained high irrespective of the amounts of mitochondria used. We also evaluated the effects of mitochondrial transfer on cells with induced mitochondrial dysfunction by treatment with oligomycin^24^ and ethidium bromide (EtBr) (Rho^0^ cells)^25^. Finally, we compared changes in mitochondrial metabolic function and the signalling pathway from dexamethasone (Dexa)-induced atrophic L6 muscle cells receiving intact mitochondria and damaged mitochondria.

## Results

### Characterization of isolated mitochondria

We first isolated mitochondria from human umbilical cord-derived mesenchymal stem cells (UC-MSCs) by differential centrifugation (Fig. S1A). Mitochondrial viability, mitochondrial purity, and mitochondrial function were then comprehensively analysed. To assess mitochondrial viability, we used the MitoTracker Red CMXRos (CMXRos) probe that stains mitochondria and its accumulation is dependent on the mitochondrial membrane potential (MMP). In other words, this probe allows for identification of viable, respiration competent mitochondria^26^. Also, the identity of endogenous mitochondria was confirmed by counterstaining with MitoTracker Green (MTG). As shown in Fig. S1B–D, isolated mitochondria were clearly stained both with MTG and CMXRos, indicating that isolated mitochondria from UC-MSCs maintained their membrane potential and were viable. The purity of isolated mitochondria was determined by assessing the presence of functional mitochondrial markers [cytochrome C oxidase (COX IV) and cytochrome c] and absence of nuclear markers [proliferating cell nuclear antigen (PCNA) and β-actin]. Western blots against COX IV and cytochrome c confirmed the presence of mitochondrial proteins in the isolated mitochondria, while PCNA and β-actin proteins were absent, confirming the high purity of the isolated mitochondria (Fig. S1E). Also, Fig. S1F shows mitochondria with reticulated morphology by electron microscopy. Finally, the ATP content of various amounts of mitochondria (0.05, 0.5 and 5 μg) increased proportionately thus confirming their functionality (Fig. S1G).

### Validation of mitochondrial transfer

**Figure 1:**
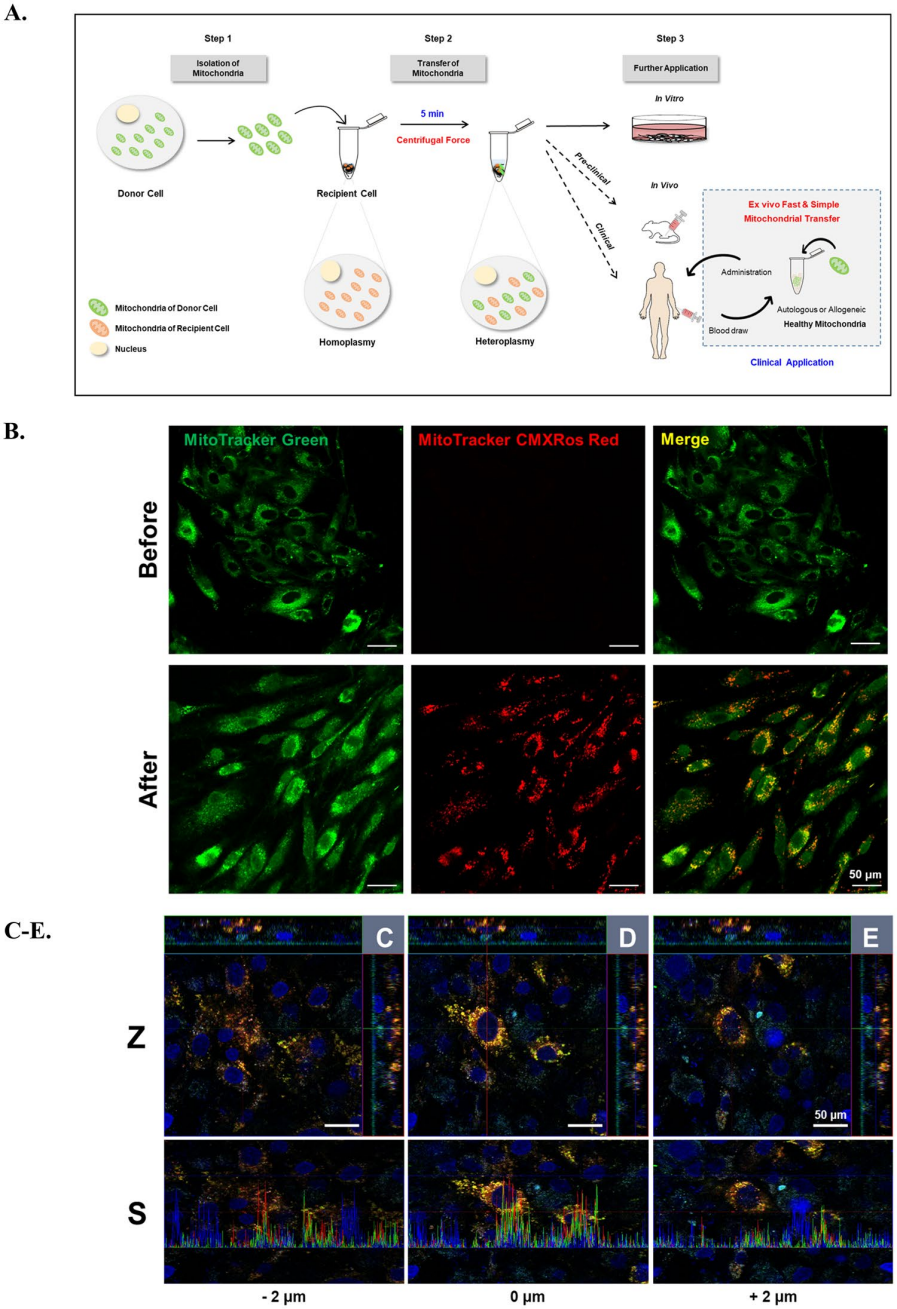
Confocal microscopic analysis of target cells following mitochondrial transfer. (**A**) Experimental scheme for mitochondrial transfer and further application. The picture was drawn by us. (**B**) Representative images of UC-MSCs co-stained with fluorescent mitochondrial dyes (MitoTracker Green and MitoTracker Red CMXRos) at 24 h after mitochondrial transfer in the before mitochondrial transfer (upper panels) and after mitochondrial transfer (lower panels). Green: endogenous mitochondria of UC-MSCs (recipient cells), red: transferred mitochondria isolated from UC-MSCs, yellow: merged mitochondria. (**C**–**E**) Three confocal sections are shown in Z-stack overlay mode. Transferred mitochondria (red) within UC-MSCs were detected in the orthogonal view (upper panels; Z) and the corresponding signal profile (lower panels; S) together with endogenous mitochondria (green). Results are from the centre of the mitochondrial network of UC-MSCs (**D**) and 2 μm below (**C**) and 2 μm above (**E**) it. Z: Z stack image-ortho analysis, S: signal profile of each section. Scale bar, 50 μm.

In order to effect transfer of isolated mitochondria into target cells, we elected on using a quick and simple centrifugation method. Figure 1A shows our experimental scheme for mitochondrial transfer and further application. Isolated intact mitochondria can be efficiently transferred into prepared recipient cells by centrifugation at 1,500 × *g* for 5 min. This condition was established through preliminary experiments assessing transfer efficiency over time and centrifugal force (Fig. S2A).

We confirmed the presence of the transferred mitochondria by confocal microscopy. As shown in Fig. 1B, exogenous mitochondria stained with CMXRos were mixed with UC-MSCs whose endogenous mitochondria were stained with MTG, and then immediately subjected to centrifugation. As expected, exogenous mitochondria were transferred into UC-MSCs (Fig. 1B) by simple centrifugation. Transferred exogenous mitochondria (red) co-localised with endogenous mitochondria (green) from UC-MSCs, indicating movement of exogenous mitochondria inside the cells as evidenced by the merged yellow staining. To further assess the localisation of internalised mitochondria, we generated a z-stack of confocal images of UC-MSCs having received exogenous mitochondria stained with CMXRos (Fig. 1C,D and E). Our results clearly show that the presence of transferred mitochondria within recipient cells, as evidenced by the merged yellow staining, confirming that exogenous mitochondria can be efficiently transferred into recipient cells by centrifugation.

### Quantification of mitochondrial transfer

**Figure 2:**
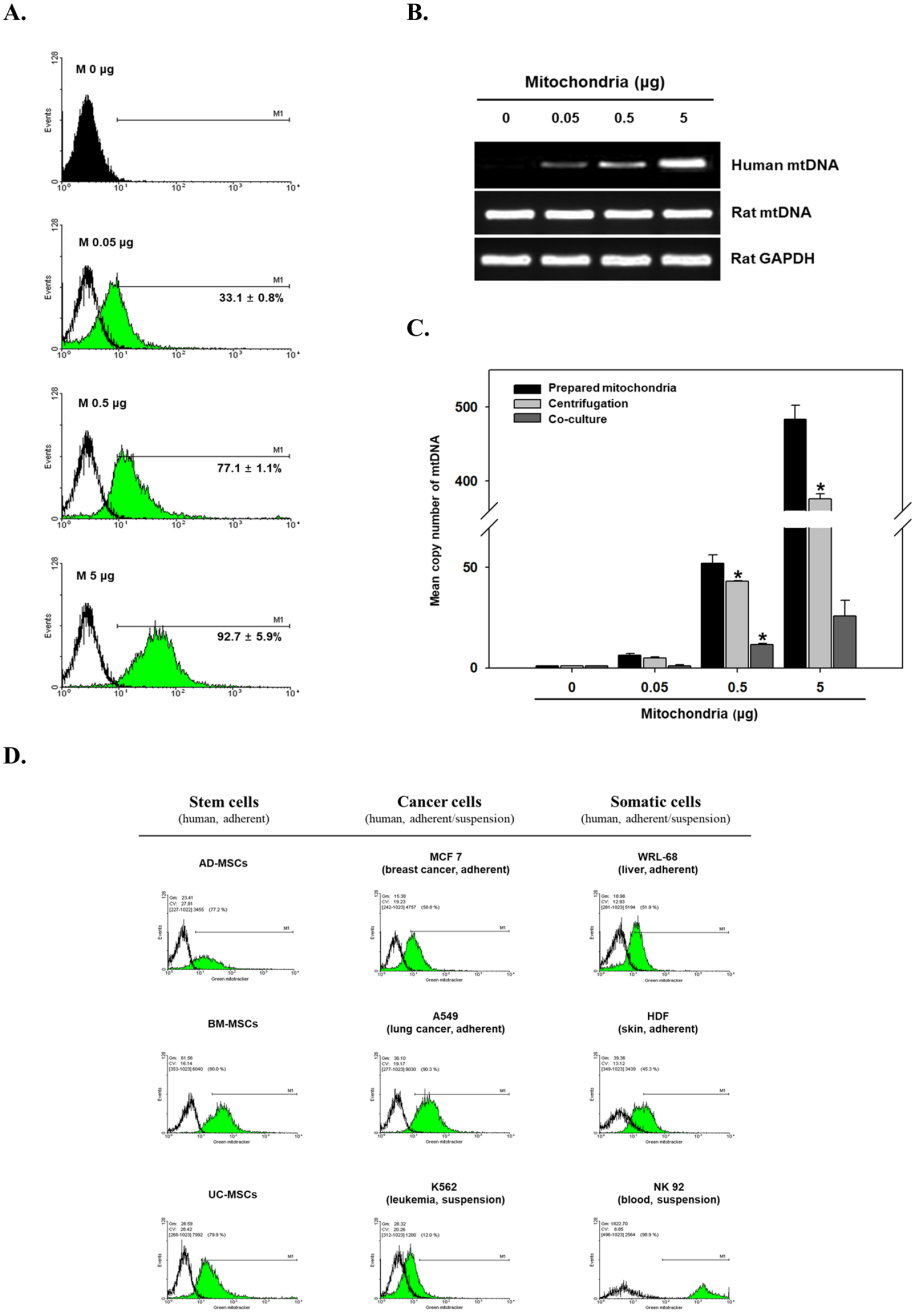
Effect of mitochondrial dose on mitochondrial delivery efficiency. (**A**) Flow cytometric analysis of MitoTracker Green fluorescence in UC-MSCs at 24 h after transfer of various amounts of mitochondria (expressed as μg of protein). (**B**) qPCR analysis of human mtDNA (h-mtDNA), rat-mtDNA and rat-glyceraldehyde-3-phosphate dehydrogenase (GAPDH) in rat L6 muscle cells after transfer of mitochondria isolated from UC-MSCs. (**C**) Real-time PCR analysis of the mean h-mtDNA copy number of mitochondria isolated from UC-MSCs for transfer (prepared mitochondria in bar definition), rat L6 cells after mitochondrial transfer via centrifugal force (centrifugation) and rat L6 cells after 1 day of co-culture (co-culture). Relative mtDNA copy numbers normalised to the GAPDH level are shown. All values are mean ± SEM. N = 3, *P < 0.05 vs. normal UC-MSCs (0 μg). (**D**) Comparison of the transfer efficiency, as determined by the intensity of green fluorescence, by various cell types upon centrifugation for 5 min at 1,500 × *g*. The information of all cell lines were represented in Supplementary information section.

To quantify the efficiency of mitochondrial transfer, flow cytometry (FACS) and PCR analyses were performed. Mixtures of UC-MSCs with exogenous mitochondria were subjected to centrifugation and then to FACS. The results of FACS analysis showed that the uptake of exogenous mitochondria increased with the amount of mitochondria used; 33.1 ± 0.8%, 77.1 ± 1.1% and 92.7 ± 5.9% of cell exhibited green fluorescence following transfer of 0.05, 0.5 and 5 μg of mitochondria into UC-MSCs, respectively (Fig. 2A). To further support the efficiency of our centrifugation transfer method, mitochondria (0.05 μg of protein) were passively transferred into UC-MSCs by co-incubation at 37 °C without centrifugation (Fig. S2B). Different concentrations of Pluronic F-68 (PF-68), which increases the fluidity of cell membranes^27^, were used during passive transfer. Nevertheless, the passive transfer efficiency of mitochondria was incomparable to that of the centrifugation method (Fig. S2B). Additionally, we tested the effect of UC-MSCs pretreatment with PF-68 prior to mitochondrial transfer by centrifugation. In contrast with the passive transfer results, increases in transfer efficiency were observed along with membrane permeability modulation of recipient cells by pre-treatment with PF-68 prior to centrifugation. Especially, the highest transfer efficiency was obtained after pretreatment with 20 mg/mL PF-68 for 2 hours, compared to centrifugation alone (90.7 ± 8.8% vs. 34.3 ± 1.8%) (Fig. S2C). Taken together, our results suggest that mitochondrial transfer by centrifugation is very effective. It also shows that controlling membrane permeability enhances transfer efficiency.

Next, after transfer of exogenous mitochondria isolated from UC-MSCs into L6 cells, the quantity of mtDNA of exogenous mitochondria in L6 cells was evaluated by quantitative PCR (qPCR) analyses. Specific PCR primers for UC-MSCs (human) and L6 cells (rat) were used. As expected, the amount of human-mtDNA (h-mtDNA) significantly increased in L6 cells proportionally to the amount of mitochondria transferred, whereas rat-mtDNA did not change, indicating that exogenous mitochondria caused the increase in mtDNA (Fig. 2B). The mean h-mtDNA copy number in L6 cells after transfer and of prepared mitochondria before transfer was as follows: 4.9 ± 0.6 vs. 6.2 ± 1.0 for 0.05 μg, 49.1 ± 0.3 vs. 52.1 ± 4.3 for 0.5 μg and 375.6 ± 7.2 vs. 483.5 ± 19.0 for 5 μg (Fig. 2C). Also, we compared the efficiency of our mitochondrial transfer protocol with that of conventional method. As shown in Fig. 2C, the h-mtDNA copy number was higher in mitochondrial transfer via centrifugation than that via passive transfer by co-culture. These results show that mitochondria are efficiently transferred into target cells through centrifugation alone. Thus, the results from centrifugal transfer indicate that mitochondria can pass through the cell membrane more easily than using the passive transfer approach.

Next, we tested mitochondrial transfer efficiency into various cells by FACS analysis. As shown in Fig. 2D, we studied three groups of recipient cells (stem cells, cancer cells and somatic cells). Cells were divided in two types (adherent and suspension cells), and prepared mitochondria from UC-MSCs (donor cells) were transferred into each type of cells. FACS analyses revealed an increase in green fluorescence following transfer of mitochondria into all cell types. Taken together, these results suggest that centrifugal force alone effectively results in mitochondrial transfer into cells *in vitro*, regardless of cell type.

### Evaluation of the effect of mitochondrial transfer on normal cells

Normally, the level of oxidative stress in cells plays a significant role in the activation of signaling pathways central to the control of energy balance, mitochondrial dynamic, cell proliferation and apoptosis. Thus, using UC-MSCs, we monitored intracellular changes in ROS production, ATP content, cell proliferation, cytosolic cytochrome c content, the expression of mitochondrial dynamic-related markers, and oxygen consumption rate (OCR) (Fig. S3 and Supplementary results). These results show that exogenous mitochondria can be transferred into target cells via centrifugation without causing intracellular damage and potentially increasing the ATP content and improving metabolic activity.

### Functional recovery of dysfunctional cells following mitochondrial transfer

We next investigated the effects of mitochondrial transfer on mitochondrial dysfunction-induced cells by treatment with oligomycin^24^ and on mtDNA-depleted cells (Rho^0^) by treatment with EtBr^25^. It is important to establish that damaged cell or stressed cell mitochondria still can generate ATP or are able to maintain their membrane potential by hydrolysing glycolytic ATP. The addition of oligomycin depolarizes mitochondria forcing the cells to eventually rely on glycolytic ATP^28^.

First, UC-MSCs were treated with oligomycin to inhibit ATP synthase activity followed by mitochondrial transfer by centrifugation (Fig. S4). After 48 h, cell proliferation and ATP content of UC-MSCs increased proportionately to the amount of the mitochondria transferred (Fig. S4A and B, respectively). In addition, we investigated the MMP and mROS levels, which are measures of mitochondrial dysfunction. MMP is the most common parameter used for monitoring mitochondrial function as well as intracellular ATP content^29^. The MMP and mROS level were lower and higher, respectively, in oligomycin-treated cells than in untreated cells (Fig. S4C and D, respectively). On the other hand, 48 h after mitochondrial transfer, MMP had increased by 80% (Fig. S4C) and the level of mROS was restored to normal levels (Fig. S4D). These results were significant and proportional to the amount of mitochondria transferred (P < 0.05). Overall, our results show that mitochondrial function is restored through the transfer of exogenous mitochondria in terms of ATP production, MMP regulation and mROS production.

We also tested the expression of AMP-activated protein kinase (AMPK) and peroxisome proliferator-activated receptor gamma coactivator 1-alpha (PGC-1α) critical for energy homeostasis and adaptation to metabolic stress^30^. As expected, upon exposure to oligomycin, AMPK was activated and PGC-1α expression decreased. As shown in Fig. S4E and F, 48 h after mitochondrial transfer, AMPK activation and PGC-1α expression were restored to normal levels in a dose-dependent manner, consistent with the intracellular ATP content (Fig. S4A). These results indicate that mitochondrial function is restored in damaged cells following mitochondrial transfer.

Next, in order to investigate the role of exogenous mitochondria in mtDNA-depleted UC-MSCs (UC-Rho^0^), UC-Rho^0^ cells were generated by treatment with EtBr^25^. We first confirmed that treatment of UC-MSCs cells with 200 ng/ml EtBr for 6 weeks results in Rho^0^ cells (Fig. S5A).

Compared with non-treated UC-MSCs (N), the levels of UC-MSC-specific mtDNA (cord-mtDNA) and human-specific mtDNA (universal-mtDNA) were decreased to 3.2 ± 0.9% and 3.7 ± 0.8% in EtBr-treated UC-MSCs, respectively (Fig. S5A). The expression of h-mtDNA was not confirmed after serial-passages (Fig. S5B). In UC-Rho^0^ cells, intracellular ATP content was decreased by 60%, although this was not statistically significant (Fig. S5C). With these changes, mitochondrial staining with CMXRos after 6 weeks showed that the mitochondrial distribution in UC-Rho^0^ cells was altered, looking like a ring compared to normal UC-MSCs (Fig. S5D). In addition, treatment with EtBr effectively impaired oxygen consumption, with approximately 2.2-fold and 4.1-fold reductions in basal and maximal OCR, respectively (Fig. S5E,F and G). Also, OCR decreases were observed in ATP production and spare respiratory capacity in UC-Rho^0^ cells (Fig. S5E,H and I). These results indicate that EtBr treatment of UC-MSCs reduces their metabolic activity by controlling their respiratory capacity thus confirming the induction of UC-Rho^0^ cells.

**Figure 3:**
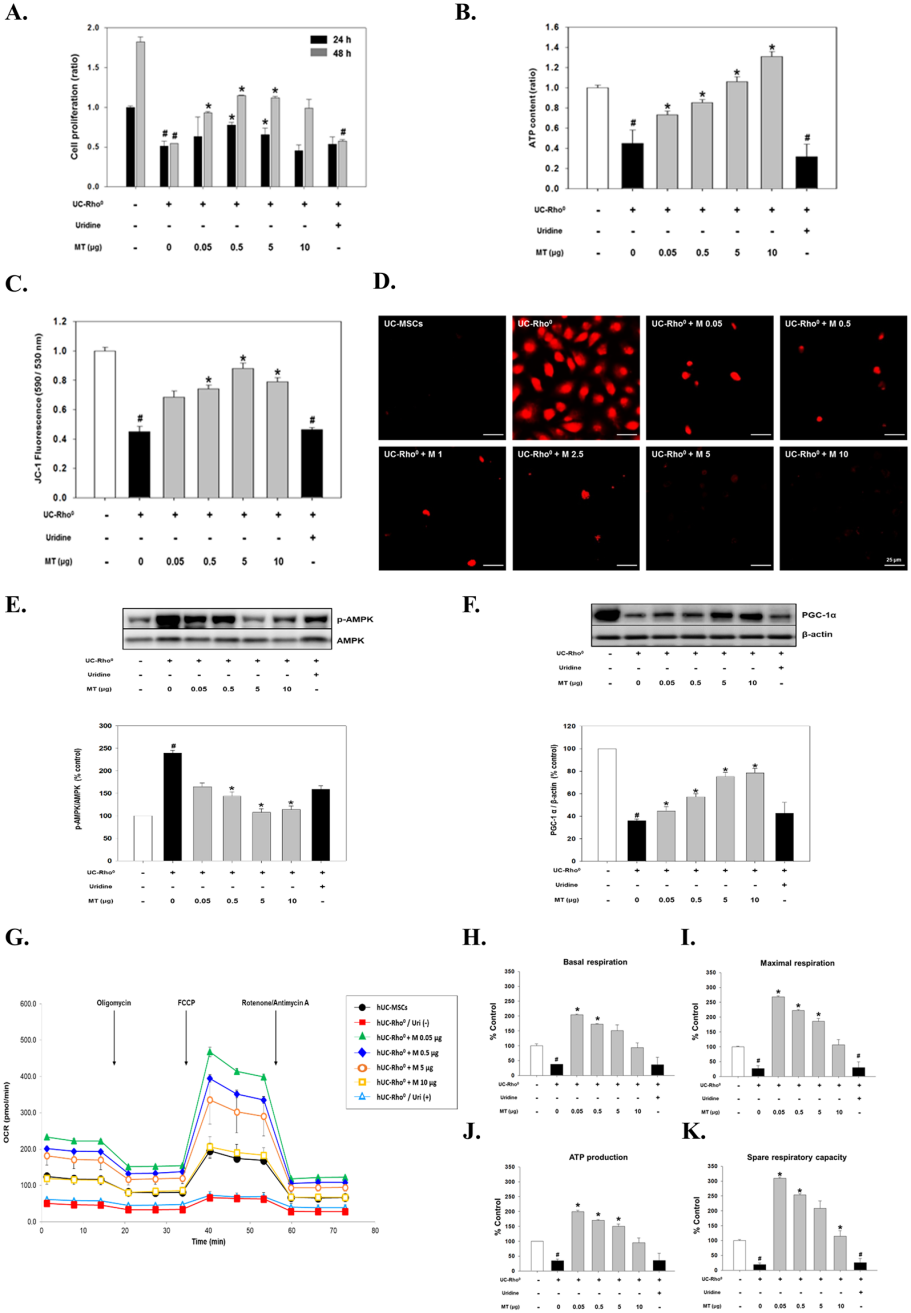
Changes in UC-Rho^0^ cell metabolism after delivery of intact mitochondria isolated from UC-MSCs. All analyses were performed 48 h after transfer of various amounts of mitochondria (expressed as μg of protein). Changes in cell proliferation (**A**), intracellular ATP content (**B**), MMP (**C**) and mROS level **(D**) were obtained and compared to the effects of mitochondrial transfer under uridine-free (−) conditions. Scale bar, 100 μm. (**E**,**F**) Immunoblot analysis of AMPK (**E**) and PGC-1α (**F**). Representative western blots and quantified expression levels (normalised to β-actin expression) are shown. (**G**) OXPHOS activity. The OCR (pmol/min) was measured in triplicate in three experiments. The OCRs of basal respiration (**H**), maximal respiration (**I**), ATP production (**J**) and spare respiratory capacity (**K**) were determined in UC-MSCs or UC-Rho^0^ cells. Cells cultured with uridine were used as control since it is needed to sustain viability of UC-Rho^0^ cells. All data represent the mean ± SEM. N = 3, *P < 0.05 vs. UC-Rho^0^ cells, ^#^P < 0.05 vs. UC-MSCs. The grouping of blots were obtained from different parts of the same gel. Full length image of results by Western blot were represented in Fig. S7.

To confirm the functionally recovery mediated by mitochondrial transfer, isolated mitochondria were transferred into UC-Rho^0^ cells, and then cell proliferation and ATP content were analysed (Fig. 3A and B). After 48 h, cell proliferation was significantly increased in a dose-dependent manner when up to 5 μg of mitochondria was transferred (Fig. 3A). The intracellular ATP content was also significantly increased in a dose-dependent manner (1.62-fold, 1.89-fold, 2.3-fold and 2.9-fold using 0.05, 0.5, 5 and 10 μg of mitochondria, respectively) (Fig. 3B). The effect of mitochondrial transfer was also evaluated by analyses of MMP and mROS. As shown in Fig. 3C, MMP was reduced by more than 50% in UC-Rho^0^ cells, and it was significantly restored to almost normal levels after mitochondrial transfer. Also, the increased mROS level in UC-Rho^0^ cells, as indicated by the fluorescence intensity of MitoSOX Red, was markedly reduced 48 h after mitochondrial transfer (Fig. 3D).

Interestingly, Rho^0^ cells need specific supplementation with uridine to sustain their viability. However, uridine did not affect cell proliferation (Fig. 3A), ATP production (Fig. 3B), and MMP maintenance (Fig. 3C). Significantly, despite the absence of uridine, our results suggest that mitochondrial functions were restored in a dose-dependent manner after mitochondrial transfer in terms of cell proliferation, ATP production, MMP maintenance and ROS production.

Activation of AMPK, which was highly elevated in UC-Rho^0^ cells, was markedly reduced by mitochondrial transfer (1.7-fold, 2.2-fold and 2.0-fold using 0.5, 5 and 10 μg of mitochondria, respectively) (Fig. 3E). Furthermore, expression of PGC-1α significantly increased to normal levels 48 h after mitochondrial transfer (44.5 ± 4.2%, 57.3 ± 2.7%, 75.3 ± 3.7% and 78.5 ± 3.9% using 0.05, 0.5, 5 and 10 μg of mitochondria, respectively, compared with 35.9 ± 1.7% in UC-Rho^0^ cells) (Fig. 3F), consistent with ATP production (Fig. 3B) and MMP maintenance (Fig. 3C). These results indicate that mitochondrial function of UC-Rho^0^ cells is restored following mitochondrial transfer.

Next, the metabolic activity of UC-Rho^0^ cells was investigated (Fig. 3G). Unlike untreated UC-Rho^0^ cells, mitochondrial transfer effectively improved oxygen consumption of the basal and maximal OCR, respectively (Fig. 3H and I). Also, increases in OCR were observed in ATP production and spare respiratory capacity after mitochondrial transfer (Fig. 3J and K). Unlike normal UC-MSCs, UC-Rho^0^ cells showed a recovery effect inversely proportional to the amount of mitochondria transferred. These results, similar to results from other studies^31^, showed that transfer of large amounts of mitochondria (>5 μg) into damaged cells had little or no effect while a rather significant increase in basal OCR (Fig. 3H), maximal OCR (Fig. 3I), ATP production (Fig. 3J), and spare respiratory capacity (Fig. 3K) were observed with lower concentration of mitochondria (0.05 and 0.5 μg).

### Mitochondrial transfer prevents mitochondrial dysfunction and metabolic alterations in cultured myotubes with Dexa-induced muscle atrophy

We selected a concentration of 1 μM Dexa as an effective concentration to induce muscle atrophy in L6 cells, as previously reported^32^. We found ATP deprivation and generation of mROS after Dexa treatment at 24 h (Fig. S6A and B, respectively). Therefore, all induction of muscle atrophy were performed using these conditions.

**Figure 4:**
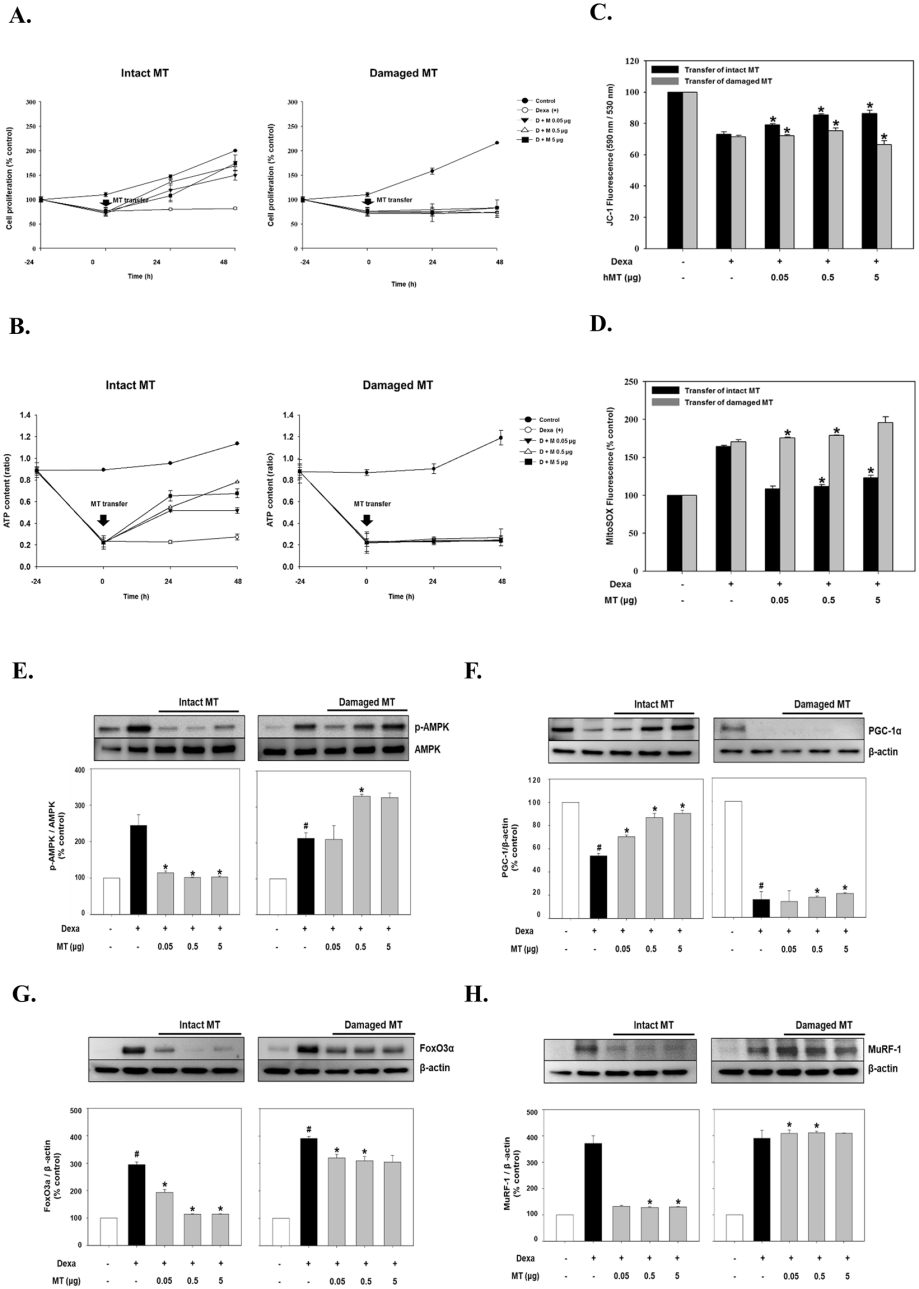
Metabolic changes in Dexa-treated atrophic L6 muscle cells after transfer of intact or damaged mitochondria. All analyses were performed 48 h after transfer of various amounts of mitochondria (expressed as μg of protein). Mitochondria were prepared from UC-MSCs grown under normal conditions (intact MT) or treated with oligomycin (damaged MT). Changes in cell proliferation (**A**), intracellular ATP content (**B**), MMP (**C**) and mROS level (**D**) were obtained and compared among the groups. (**E**–**H**) Immunoblot analysis of AMPK (E), PGC-1α (**F**), FoxO3α (**G**) and MuRF-1 (**H**). Representative western blots and quantified expression levels (normalised to β-actin expression) are shown. All data represent the mean ± SEM. N = 3, *P < 0.05 vs. Dexa (+) group, ^#^P < 0.05 vs. UC-MSCs. The grouping of blots were obtained from different parts of the same gel. Full length image of results by Western blot were represented in Fig. S7.

In the current study, we aimed to further explore whether a protective effect could be achieved by transfer of healthy exogenous mitochondria (denoted as intact MT), we added the transfer condition with damaged mitochondria (denoted as damaged MT) exposure to oligomycin, ATP synthase inhibitor (Fig. 4). After 48 h, cell proliferation was significantly increased in a dose-dependent manner after transfer of intact MT (Fig. 4A). ATP content increased in a time-dependent manner and was higher after transfer of intact MT (0.5 μg) compare with Dexa-treated cells not subjected to mitochondrial transfer [Dexa (+)] (Fig. 4B). Also, the MMP, which was reduced by 30% by treatment with Dexa, was significantly restored proportionately to the amount of mitochondria transferred compare with the Dexa (+) group (Fig. 4C). The mROS level was reduced to normal levels after mitochondrial transfer compared to the Dexa (+) group (Fig. 4D). In contrast, no recovery was observed when damaged MT were transferred looking at cell proliferation, ATP production, MMP regulation and mROS production. These results suggest that it is important to transfer intact mitochondria into cells for recovery of mitochondrial function.

Functional recovery mediated by mitochondrial transfer was also assessed by analysing the activation of AMPK and PGC-1α (Fig. 4E and F). 48 h after mitochondrial transfer, AMPK activation, which was increased up to 2.5-fold by treatment with Dexa, was restored to almost normal levels by transfer of intact MT compared with the Dexa (+) group (Fig. 4E). Also, PGC-1α expression which was reduced by treatment with Dexa was significantly restored at 48 h proportionately to the amount of mitochondria transferred (70.4 ± 1.4%, 86.8 ± 3.6% and 90.4 ± 2.8% following transfer of 0.05, 0.5 and 5 μg of mitochondria, respectively, compared with 53.9 ± 2.2% for the Dexa (+) group (Fig. 4F). Likewise, transfer of damaged MT did impact the expression of AMPK and PGC-1α, which is consistent with ATP production (Fig. 4B). These results also show that the transfer of intact mitochondria is very important for functionally recovery.

To further investigate whether mitochondrial transfer contributes to the recovery of atrophic signaling in muscle cells, we studied the accumulation of FoxO3α and the expression of muscle atrophy-related protein muscle ring finger-1 (MuRF-1). We observed a significant down-regulation of FoxO3α and MuRF-1 in cells receiving MT compared with the Dexa (+) group (Fig. 4G and H). Interestingly, upon transfer of 0.5 or 5 μg of intact MT, we observed the best efficiency of recovery as evidenced by a dramatic decrease in FoxO3α (113.4 ± 1.5% and 114.1 ± 1.1%, respectively) and MuRF-1 (128.4 ± 2.8% and 129.8 ± 2.1%, respectively) expression to levels similar to that of untreated cells (Fig. 4G and H, respectively). By contrast, transfer of damaged MT failed to improve any of the parameters related to mitochondrial function or atrophic signalling in muscle cells (Fig. 4).

## Discussion

This study reports a method to transfer mitochondria into cells. Our protocol provides quicker and simpler procedures than previously reported ones. The prepared mitochondria were transferred into target cells via centrifugation at 1,500 × *g* for 5 min without additional incubation. The advantages of our protocol are as follows: First, exogenous mitochondria can be transferred regardless of cell type or species. Second, mitochondrial transfer by centrifugation improved mitochondrial function such as intracellular ATP content and metabolic activity. Third, transfer of healthy exogenous mitochondria efficiently prevented Dexa-induced muscle atrophy, mainly by improving mitochondrial function and blocking the atrophic signalling pathway. Collectively, we established a method to simply deliver exogenous mitochondria into target cells via centrifugal force. This method restored the function of damaged cells.

Mitochondria are relatively large, negatively charged and therefore difficult to transfer into cells. However, many studies reported the successful transfer of intact mitochondria into cultured cells. These previous studies used Pep-1^21^, magnetic nanoparticles^22^, or other additives^23^ to enhance the efficiency of mitochondrial delivery into target cells. Furthermore, large amounts of mitochondria were needed and additional cultivation had to be performed. Chang *et al*. reported that mitochondrial functions of cells (cell viability and mitochondrial biogenesis) with mutant mtDNA could be restored by transferring Pep-1-conjugated mitochondria^21^. However, non-internalised Pep-1-labelled mitochondria were still present in the culture medium after 2 days of incubation. This was attributed to the large size of mitochondria (about 500–1,000 nm), which prevented them from entering the cells. Caicedo *et al*. studied the metabolic function of cancer cells by transferring mitochondria isolated from mesenchymal stem cells into cultured cancer cells^23^. Cancer cells were plated, mitochondria were added and cultures were centrifuged twice. Co-culture was then performed for 24 h to transfer mitochondria. Macheiner *et al*. improved delivery efficiency by treating cultured cells with mitochondria labelled with anti-TOM22 magnetic beads and placing them on magnetic plates^22^. We efficiently delivered mitochondria by mixing them with target cells in a small tube and then centrifuging the sample for 5 min. This method does not require any additional incubation process. Furthermore, a small amount of mitochondria could be efficiently transferred into target cells. Consequently, a sufficient amount of mitochondria can be obtained from a small number of cells or a small amount of tissue.

While it is possible deliver mitochondria into cultured cells via incubation, the mechanism of mitochondrial entry into cells remains controversial. We demonstrated that centrifugal force causes mitochondria to penetrate the cell membrane and enter cells. Moroz (1984) theorised that centrifugal force can mediate cell-to-cell transfer of nuclear and cytoplasmic organelles^33^. Thus, we hypothesised that membrane permeability is the main factor influencing entry of mitochondria into cells. Therefore, we investigated whether the efficiency of mitochondrial delivery improved as membrane permeability increased. PF-68 is a non-ionic surfactant that enhances cell membrane permeability^27^. Target cells were treated with various amounts of PF-68 for different durations without eliciting cytotoxic effects, and the efficiency of mitochondrial delivery was compared before and after centrifugation (Fig. S2D and E). As expected, the amount of mitochondria penetrating the cell membrane increased as cell permeability increased. However, even when cell permeability increased, mitochondria were not transferred into cells without centrifugation. We also confirmed that mitochondrial transfer was possible irrespective of the donor or recipient cell type (Fig. 2D). The difference in mitochondria transfer efficiency between cells under the same conditions seems to be the result of the difference in cell-specific membrane fluidity^34^. Based on these results, we were convinced that centrifugal force alone could deliver mitochondria into cells *in vitro*, regardless of the cell type.

Under normal physiological conditions, maintenance of the proper balance between mROS and ATP production is crucial to produce the energy required by the cell^35^. However, ROS produced in mitochondria under pathologic conditions cause oxidative damage to the inner membrane where mitochondrial respiratory chain enzymes are located^36,37^. In addition, a decreased level of mitochondrial respiratory chain enzymes associated with ATP production, as well as a reduced MMP, may cause oxidative damage to proteins and mtDNA, leading to accumulation of mtDNA mutations^36,37^. In this regard, we sought to validate the efficacy of exogenous mitochondrial delivery *in vitro*. We observed changes in mitochondrial-related functions after delivery of various quantities of mitochondria into normal cells or cells in which mitochondrial dysfunction had been induced. In normal cells, mitochondrial delivery via centrifugal force did not cause intracellular damage, increase in oxidative stress (intracellular ROS and mROS) or apoptosis, as assessed by cytochrome c release from mitochondria to the cytosol (Fig. S3). On the other hand, transfer of intact MT restored mitochondrial function of cells in which mitochondrial ATPase was inhibited by oligomycin or mtDNA was deleted by EtBr, as evidenced by an increase in their MMP, a decrease in the mROS level and increases in ATP production and respiration rate (Fig. S4, Fig. 3, respectively). These results suggest that mitochondria delivered by centrifugal force can enhance various intracellular functions without causing intracellular damage, and may increase the ATP content and improve metabolic activity. However, further studies are needed to confirm that exogenous mtDNA delivered by our method is stable and persistent in the recipient Rho^0^ cell line.

Mitochondria are the main cellular energy-producing organelles and their dysfunction leads to an insufficient energy supply and activation of several intracellular signalling pathways, including AMPK activation, autophagy (mitophagy) and/or apoptosis. ATP deprivation usually promotes robust AMPK activation, which is regulated by the ratio of intracellular AMP to ATP and leads to mitochondrial biogenesis by phosphorylating PGC-1α or autophagy^38–40^. Overall, our results demonstrate that transferred exogenous mitochondria regulate PGC-1α-mediated mitochondrial biogenesis and AMPK activation by increasing the intracellular ATP content of functionally impaired cells.

Another important finding of the current study is that transfer of healthy exogenous mitochondria efficiently prevented Dexa-induced muscle atrophy, mainly by improving mitochondrial function and blocking the atrophic signalling pathway (Fig. 4). The energy sensor AMPK can directly regulate FoxO3 via phosphorylation^41^, and the AMPK/FoxO3 pathway participates in muscle atrophy induced by enforced mitochondrial fission^5^. Here, we provide *in vitro* evidence that Dexa robustly induced respiratory dysfunction, resulting in intracellular ATP depletion and AMPK activation, which subsequently activated the FoxO3/Atrogene pathway. Transfer of exogenous mitochondria can regulate this signalling pathway by increasing intracellular ATP content. The metabolic function of atrophic cells was not restored by delivery of mitochondria whose function had been impaired by oligomycin. Taken together, these findings suggest that mitochondrial dysfunction and ATP depletion play an important role in the pathogenesis of Dexa-induced muscle atrophy, consistent with other studies^42^. To improve muscle atrophy, the isolated mitochondria should be intact, viable and respiration-competent. Our data provide new insight into the involvement of mitochondria in Dexa-induced muscle atrophy and suggest a promising strategy involving transfer of mitochondria to improve mitochondrial function. Furthermore, these findings provide a basis for further mitochondria-related investigations into the rescue of atrophy and the restoration of muscular function.

It is important to screen and acquire mitochondria from healthy sources since isolated mitochondria may be functionally distinct depending on their origin. In addition, the effects of autologous, allogeneic and xenogeneic mitochondria on cell metabolism are not fully understood. Therefore, further studies should be conducted to determine the effects of mitochondria from different origins on the function of various cell types. Isolated mitochondria should remain healthy for a long time because it is not feasible to cultivate cells each time mitochondrial transfer is performed. mtDNA can be easily damaged by the external environment, which makes it difficult to maintain mitochondria in an intact state. Therefore, it is most cost-effective and safest to keep large amounts of mitochondria at once and use them immediately when needed. However, long-term preservation of isolated mitochondria remains challenging.

The metabolic functions of adult stem cells, immune cells, or somatic cells decrease with aging. Recently immune cell therapy to boost immunity or to treat cancer become popular^43,44^. Conventional immune cell therapy requires large amount of effective cells. In our studies, we observed that immune cells with exogenous mitochondria show almost two times higher cytotoxicity against cancer cells (unpublished data), suggesting that *ex vivo* manipulation of immune cells by transferring exogenous mitochondria could provide a better clinical solution to treat cancer patient.

## Materials and Methods

### Cell culture

This study was performed with the approval from the Institutional Review Board (IRB) of CHA University (Seongnam, Korea; IRB No. 201511-BR-022-02) and the protocol used in the study were approved by the IRB from the CHA General Hospital. All information pertaining to subjects and all human samples were used in compliance with Korean legislation, and all human participants provided informed written consent^45^. UC-MSCs were isolated and cultured as described previously^32^. Briefly, UC-MSCs were grown in Minimum Essential Medium Eagle Alpha Modification (α-MEM; Hyclone Laboratories Inc., Logan, UT) supplemented with 10% foetal bovine serum (FBS; Hyclone), 100 μg/ml streptomycin plus 100 IU/ml penicillin (P/S; Hyclone) and 10 ng/ml basic fibroblast growth factor (CHA Meditech Co., Daejeon, Korea). Cells were passaged when they approached a predetermined confluency (80–90%). UC-MSCs were used at passage #5–7 for all experiments. The L6 rat myoblast cell line (CRL-1458) was purchased from the Animal Type Culture Collection (Manassas, VA). These cells were grown in Dulbecco’s modified Eagle minimal essential medium (DMEM; Hyclone) supplemented with 10% FBS and 1% P/S. L6 cells were used at passage #6–10 for all experiments.

### Isolation and labelling of mitochondria

The Supplementary information section contains all protocols for mitochondrial isolation and labelling.

### Mitochondrial transfer into recipient cells

Prior to mitochondrial transfer, recipient cells prelabelled with MitoTracker Green were harvested from culture flasks, and 1 × 10^5^ cells were transferred to a microcentrifuge tube. Cells were suspended in 100 μl of PBS and kept on ice for transfer. The amounts of mitochondria cited in the text refer to the amounts of donor cell mitochondria (μg of protein) per 1 × 10^5^ recipient cells. The mitochondrial suspension (in 10 μl of PBS) was added slowly to each tube of recipient cells suspended in 100 μl of PBS. The microcentrifuge tubes were centrifuged at 1,500 × *g* for 5 min at 4 °C. Cells were then rinsed twice with PBS and imaged or lysed for further testing.

To compare the efficiency of mitochondrial transfer, the same preparations of mitochondria were transferred into 1 × 10^5^ recipient cells by co-culture. L6 cells were seeded at a density of 5 × 10^5^ cells/well in a 6-well plate for 24 h before mitochondrial transfer. The prepared mitochondria were added to the wells and then placed in a 37 °C incubator. The next day, cells were harvested for further testing.

### FACS analysis

All flow cytometric analyses were performed using a FACSCalibur system (BD Biosciences, San Jose, CA). To monitor mitochondrial transfer, cells were washed with PBS, harvested after transfer of mitochondria prelabelled with MitoTracker Green and resuspended in 500 μl of PBS. MitoTracker Green fluorescence was detected using a 488 nm laser and a 530/30 nm filter.

### PCR analysis

Total genomic DNA (gDNA) was isolated from mitochondria or cells using a NucleoSpin Tissue kit (Marcherey-Nagel, Dueren, Germany) following the manufacturer’s instructions. The following conditions were used for general PCR: pre-denaturation at 95 °C for 5 min; 25 cycles of denaturation at 95 °C for 30 s, annealing at 58 °C for 30 s and extension at 72 °C for 30 s; and a final extension at 72 °C for 5 min. PCR products were visualised by electrophoresis on a 1.5% (w/v) agarose gel stained with EtBr. Gel images were analysed with a ChemiDoc™ XRS + System (Bio-Rad Laboratories, Hercules, CA).

Real-time quantitative PCR were performed in MicroAmp optical 96-well reaction plates using a StepOnePlus Real-Time PCR System (Applied Biosystems, Foster City, CA). mtDNA expression was quantified using a SYBR Green assay on an iCycler Optical System (Applied Biosystems). PCR was performed in a final volume of 20 μl per reaction with a mixture of gDNA (1 μl), SYBR Green PCR Master Mix (10 μl) and sense and antisense primers (5 pM each) corresponding to UC-MSCs-specific mtDNA (cord-mtDNA) (sense: 5′-tgc cag cca cca tga ata tt-3′, antisense: 5′-ggt ggg tag gtt tgt tgg-3′), human-specific mtDNA (universal-mtDNA) (sense: 5′-tta act cca cca tta gca cc-3′, antisense: 5′-gag gat ggt ggt caa ggg a-3′) and human nuclear DNA (sense: 5′-aca caa ctg tgt tca cta gc-3′, antisense: 5′-cca act tca tcc acg ttc a-3′).

### Fluorescence image analysis

A TCS SP5 II confocal microscope (Leica, Heidelberg, Germany) equipped with 10 × and 20 × numerical aperture objectives was used to track mitochondrial transfer. Digital images were acquired using Leica LAS AF Software, version 2.6 (Leica Microsystems, Mannheim, Germany).

### Cell proliferation assay

Cell proliferation was determined under serum-free conditions using the WST-1-based, colorimetric Cyto X Cell Viability Assay Kit (LPS solution, Daejeon, Korea). UC-MSCs were seeded into 96-well culture plates (SPL, Life Sciences Co., Pocheon, Korea) at a density of 1 × 10^4^ cells/well. After 24 h, non-adherent cells were washed away with Dulbecco’s PBS (Hyclone), and the medium was replaced with fresh serum-free α-MEM. At the indicated time points (24 and 48 h), 20 μl of WST-1 solution was added to each well (final dilution = 1: 10) and the reaction mixture was incubated at 37 °C for 2 h. Absorbance at 450 nm was measured using an Epoch spectrometer/microplate reader (BioTek Inc., Winooski, VT).

### ATP determination

ATP was measured with a CellTiter-Glo luminescence kit (Promega, Madison, WI), which generates a luminescent signal proportional to the amount of ATP. Opaque-walled 96-well plates containing culture media (50 μl), cell lysate (50 μl) or isolated mitochondria (in 50 μl of PBS) were prepared followed by addition of 50 μl of CellTiter-Glo luminescence test solution and incubated for 30 min at room temperature. Luminescence signals were determined using a luminescence microplate reader.

### MMP measurement

To monitor mitochondrial health, JC-1 (Invitrogen) was used to assess the MMP after mitochondrial transfer. At 48 h after mitochondrial transfer, UC-MSCs or UC-Rho^0^ cells were incubated with JC-1 (1 μM) for 30 min at 37 °C. JC-1 accumulates in mitochondria in a MMP-dependent manner, as indicated by a shift in fluorescence emission from green (excitation 485 nm/emission 516 nm) to red (excitation 579 nm/emission 599 nm). The MMP was determined by the fluorescence ratio, as measured with a fluorescence microplate reader.

### Measurement of mROS

mROS in cells were measured with MitoSOX Red, a mitochondrial superoxide indicator (Invitrogen). Cells were seeded in a 24-well plate (1 × 10^5^ cells/well) after mitochondrial transfer and cultured for 48 h in growth medium. When they reached 80–90% confluency, cells were washed once with Hank’s Balanced Salt Solution (Hyclone) and then treated with 1 µM MitoSOX Red at 37 °C in 5% CO_2_ for 30 min. The level of mROS was determined using a fluorescence microplate reader with excitation and emission wavelengths of 510 and 528 nm, respectively.

### Extracellular flux analysis (Seahorse)

The high-throughput XF Extracellular Flux Analyzer (SeaHorse Bioscience) was used to measure the OCR (pmol/min) in recipient cells, which reflects the rate of mitochondrial respiration. All measurements were performed at 24 h after transfer of mitochondria into UC-MSCs or UC-Rho^0^ cells. Measurements was performed in XF media (non-buffered DMEM) supplemented with 2.5 mM glucose, 2 mM L-glutamine and 1 mM sodium pyruvate under basal conditions and following treatment with the following mitochondrial inhibitors: 2 μM oligomycin, 1 μM FCCP and 0.5 μM rotenone/antimycin A. The basal respiration rate was calculated as the difference between the basal OCR and the OCR after inhibition of mitochondrial complex 1 and 3 with rotenone and antimycin A, respectively. The maximum respiration rate, indicative of the maximum level of electron transport and substrate oxidation in these cells, was measured following the addition of the uncoupler FCCP (uncoupled rate). All OCR were normalised by protein amount obtained each well after OCR analysis.

### Western blot analysis

Cells were lysed with RIPA buffer containing a protease inhibitor cocktail (Roche Diagnostics). The concentration of the total protein extract was evaluated by measuring absorbance at 595 nm with an ELISA microplate reader. Protein samples were separated by 10% sodium dodecyl sulphate-polyacrylamide gel electrophoresis (SDS-PAGE). Separated proteins were transferred to a nitrocellulose membrane (Whatman, Dassel, Germany) for 3 h at 4 °C. Then, the membrane was blocked by incubation with 3% BSA (Bio Basic Inc., Ontario, Canada) prepared in PBS containing 0.1% Tween-20 (PBS-T) at room temperature. The membrane was incubated overnight at 4 °C with each of the following primary antibodies: anti-AMPKα (#2532), anti-phospho-AMPKα (#2535) and anti-FoxO3α (#2497), anti-LC-3B (#2775) (all from Cell Signaling Technology, Beverly, MA), anti-cytochrome c (sc-13156), anti-PGC-1α (sc-13067), anti-PCNA (sc-56), anti-MuRF-1 (sc-27642) and anti-β-actin (sc-47778) (all from Santa Cruz Biotechnology Inc., Santa Cruz, CA), anti- PINK1 (NBP2-36488) (from Novus Biologicals) and anti-COX IV (ab33985), anti-Mfn-2 (ab56889) and anti-DRP-1 (ab56788) (all from Abcam Inc., Cambridge, MA). After washing with PBS-T, the blots were incubated with the following horseradish peroxidase-conjugated secondary antibodies: goat anti-rabbit (1:5,000; Santa Cruz; sc-2004) and goat anti-mouse (1:5,000; Santa Cruz; sc-2005). Target proteins were visualised by enhanced chemiluminescence (ECL component of Pierce Clarity and Western ECL Substrate, Bio-Rad Laboratories) and detected with a LAS-4000 imager (Fujifilm Inc., Tokyo, Japan).

### Statistical analysis

All statistical analyses were performed using Sigmaplot 11.0 Software (Systat Software, San Jose, CA). Significant differences between groups were evaluated by a one-way analysis of variance followed by a post-hoc correction for multiple comparisons. Quantitative results are presented as mean ± SEM. Statistical significance was defined as P < 0.05.

### Data availability

All relevant data are within the paper.

## Electronic supplementary material

Supplementary information
