# Supplementary material for: Delivery of exogenous mitochondria via centrifugation enhances cellular metabolic function
Source: Sci Rep. 2018 Feb 20;8:3330. doi: 10.1038/s41598-018-21539-y (PMC5820364; doi:10.1038/s41598-018-21539-y)
Supplement: Supplementary file 1 — Supplementary information [file 41598_2018_21539_MOESM1_ESM.pdf]

Supplementary Information

Delivery of exogenous mitochondria via centrifugation enhances cellular metabolic function

Mi Jin Kim[1], Jung Wook Hwang[1], Chang-Koo Yun[1], Youngjun Lee[2], Yong-Soo Choi[1*]

[1]Department of Biotechnology, CHA University, Seongnam, 13488, Republic of Korea

[2]Paean Biotechnology Inc., Daejeon, 34028, Republic of Korea

Supplementary Methods

Isolation of mitochondria

Mitochondria were isolated from donor cells by differential centrifugation. The experimental flow chart for mitochondrial isolation is shown in Fig. S1A. In brief, mitochondria were prepared from 2×10^7 UC-MSCs. Cells were harvested from culture flasks, and pellets were homogenised using a disposable 1 ml syringe in SHE buffer [0.25 M sucrose, 20 mM HEPES (pH 7.4), 2 mM EGTA, 10 mM KCl, 1.5 mM MgCl2 and 0.1% defatted bovine serum albumin (BSA)] containing a protease inhibitor (Roche Diagnostics, Mannheim, Germany) and centrifuged at 1,100 x *g* for 3 min at 4°C (to remove unbroken cells and cell debris). The supernatant was then centrifuged at 12,000 x *g* for 15 min at 4°C to pellet mitochondria. The mitochondrial pellet was resuspended in 500 μl of SHE buffer and centrifuged at 20,000 x *g* for 10 min at 4°C, after which the supernatant was removed. The pellet was resuspended and centrifuged at 20,000 x *g* for 5 min at 4°C. After removal of the supernatant, the pellet was resuspended in 50 μl of PBS and kept on ice until measurements were performed. Isolated mitochondria were quantified by determining the protein concentration using a bicinchoninic acid assay (Pierce, Rockford, IL). All assays were performed with freshly isolated mitochondria.

Labelling of mitochondria

To visualise mitochondrial transfer, cells were separately labelled 24 h before mitochondrial isolation and transfer with MitoTracker Green FM or MitoTracker Red CMXRos (Molecular Probes, Invitrogen, Carlsbad, CA) according to the manufacturer's protocol. Briefly, cells were resuspended in prewarmed (37°C) staining solution containing the MitoTracker probe (300 nM) and incubated for 30 min under appropriate growth conditions. After staining, cells were washed thrice with phosphate-buffered saline (PBS) and resuspended in fresh prewarmed medium. The cells were harvested or plated according to the experiment.

Transmission electron microscopy

Isolated mitochondria (100 μg) were fixed with 2% paraformaldehyde and 2% glutaraldehyde (Electron Microscopy Sciences, Hatfield, PA) in 0.1 M cacodylate buffer (Electron Microscopy Sciences). The fixed samples were dehydrated through a series of ethanol and embedded in epoxy resin (Epon 812; Sigma-Aldrich, USA). Ultrathin sections (70 nm) were cut from the resin blocks by using a diamond knife mounted on an Ultracut (Leica, Tokyo, Japan). The sections were placed on copper grids, counter-stained with 2% uranyl acetate (Merck), and photographed

with transmission electron microscope (H-7650; Hitachi, Japan).

Cell preparation

Human adipose-derived mesenchymal stem cells (AD-MSCs; PCS-500-011, passage #7), a human breast cancer cell line (MCF-7; HTB-22, passage #20), human adenocarcinoma alveolar epithelial cells (A549; CCL-185, passage #20), a human leukemic cell line (K562; CCL-243, passage #20), a human hepatic cell line (WRL-68; CL-48, passage #9), human dermal fibroblasts (HDFs; PCS-201-012, passage #9) and a human natural killer cell line (NK-92; CRL-2407, passage #10) were purchased from the Animal Type Culture Collection (Manassas, VA). Human bone marrow-derived mesenchymal stem cells (BM-MSCs; MSC-001F, passage #7) were purchased from Stem Cell Inc. (Vancouver, Canada). To compare the transfer efficiency (%) according to the cell type (Figure 2d), all cell lines were incubated in a 37°C bath until completely thawed and then used immediately for mitochondrial transfer. The protocol for mitochondrial transfer is described in the Materials and Methods section.

Measurement of reactive oxygen species (ROS) using 2',7'-dichlorofluororescein diacetate (DCF-DA)

The fluorogenic substrate DCF-DA (Invitrogen) is a cell-permeable dye that is oxidised to highly fluorescent 2'7'-dichloroflurescein by H2O2 and can therefore be used to monitor intracellular generation of ROS. To measure ROS, cells were seeded into 24-well plates after mitochondrial transfer. After 48 h, cells were washed twice with PBS and incubated with 10 μM DCF-DA for 30 min. Intracellular ROS generation was determined using a fluorescence ELISA reader at an excitation wavelength of 485 nm and an emission wavelength of 535 nm (Molecular Devices).

Generation of Rho^0 cells

To generate UC-Rho^0 cells, UC-MSCs were treated with 200 ng/ml EtBr for 6 weeks. UC-Rho^0 cells were maintained in α-MEM supplemented with 10% FBS, 1% P/S, 0.1 mM non-essential amino acids, 1 mM sodium pyruvate and 50 μg/ml uridine (Sigma-Aldrich, St. Louis, MO). Specific supplements, such as pyruvate and uridine, were required to maintain their viability. The medium was changed every 2 days. The cells were used at passage #8–9 for further experiments. All cell lines were maintained at 37°C in 5% CO_2.

Induction of muscle atrophy

To induce muscle atrophy, L6 muscle cells were seeded in six-well culture plate (SPL; 2 x10^5 cells/well). And then cells were treated with 1 μM Dexa (Sigma-Aldrich) in a time-dependent manner (6, 12 and 24 h) as an in vitro model for muscle atrophy. After 24 h, cells were washed twice

with PBS and analyzed ATP content and mROS generation (Fig. S6A and B). Based on these results, prepared mitochondria were transferred to L6 cells treated with 1 μM Dexa to investigate the changes of intracellular function (Fig. 4).

Supplementary Results

Evaluation of the effect of mitochondrial transfer on normal cells. To assay the generation of intracellular ROS in UC-MSCs we used two fluorescent indicators (DCF-DA and MitoSOX) (Fig. S3A). DCF-DA is one of the most widely used probe for detecting intracellular H_2O_2 and oxidative stress. Oxidation by ROS yields DCF, which emits green fluorescence upon excitation at 494 nm[1]. Similarly, MitoSOX Red has been used to measure cellular and mitochondrial superoxide formation[1]. Using H_2O_2 -treated UC-MSCs as positive controls, cellular ROS production was not detected in UC-MSCs 48 h after mitochondrial transfer (Fig. S3A). In addition, the intracellular ATP content at 48 h was significantly increased in proportion to the amount of the mitochondria transferred (1.38-fold, 1.69-fold and 1.96-fold using 0.05, 0.5 and 5 μg of mitochondria, respectively) (Fig. S3B). However, unexpectedly, no significant effect on cell proliferation was observed despite an increase in ATP content and absence of ROS production (Fig. S3C).

Under normal physiological condition, cytochrome c is located in the mitochondrial intermembrane space. However, as mitochondrial ROS production increases, cytochrome c is translocated to the cytosol, which is a key step in the apoptotic process[2,3]. We evaluated the cytosolic cytochrome c content after mitochondrial transfer by immunoblotting (Fig. S3D). After 48 h after mitochondrial transfer, cytochrome c was not released into the cytosol of UC-MSCs (Fig. S3D). All in all, mitochondrial transfer by centrifugation does not lead to increased ROS production or the release of cytochrome c into the cytosol (Fig. S3A).

In addition, we examined changes in the expression of various markers that are important for regulating mitochondrial biogenesis[4], mitochondrial dynamics [mitofusin 2 (Mfn-2) and dynamin-1-like protein (DRP-1)] and degradation [PTEN-induced kinase 1 (PINK-1) and LC3B]. Unlike expectations, the expression of these markers were not observed at 48 h after mitochondrial transfer (Fig. S3E), suggesting that it is necessary to track changes of these markers at different time points.

Finally, the metabolic activity of UC-MSCs after mitochondrial transfer was investigated. Compared to normal UC-MSCs, mitochondrial transfer effectively improved oxygen consumption of the basal and maximal OCR, respectively (Fig. S3F, G and H). Also, increases in OCR were observed in ATP production and spare respiratory capacity after mitochondrial transfer (Fig. S3G, I and J). Importantly, the spare respiratory capacity, a major factor determining cell survival, increased proportionately to the amount of mitochondria transferred (Fig. S3J), indicating that exogenous mitochondria can modify the metabolic activity of UC-MSCs by regulating their respiratory capacity.

Supplementary Figure Legends

Supplementary Figure S1. Isolation of mitochondria from UC-MSCs.

(A) Schematic of the steps used to isolate mitochondria from UC-MSCs. (B–D) Confocal microscopy images of isolated mitochondria showing that their membrane potential was maintained. (B) Endogenous mitochondria of UC-MSCs were counterstained with MitoTracker Green prior to isolation. (C) Isolated mitochondria were stained with MitoTracker Red CMXRos, whose accumulation is dependent upon the MMP. (D) Overlay of (B) and (C). Scale bar, 100 μm. (E) To verify the purity of mitochondria, the enrichment of intact mitochondria was confirmed by immunoblot analysis of cytosolic and mitochondrial fractions using antibodies against COX IV (a mitochondrial marker), cytochrome C (a mitochondrial marker), PCNA (a nuclear marker) and β-actin (a cytosolic marker). (F) Transmission electron microscopy of isolated mitochondria. Scale bar, 1,000 nm. (G) The ATP contents of the specified amounts of isolated mitochondria (μg of protein) were measured by a chemiluminescence assay. All values are shown as mean ± SEM. N=3. *P < 0.05 vs. normal UC-MSCs (0 μg). The grouping of blots were obtained from different gel, respectively. Full length image of results by Western blot were represented in Fig. S7.

Supplementary Figure S2. Verification of mitochondrial delivery using centrifugal force.

(A) representative result showing the ratio of green fluorescence determined by flow cytometry following mitochondrial transfer (0.05 μg of protein) according to the centrifugation duration (5, 10 and 15 min) and centrifugal force (500, 1,000, 1,500 and 2,000 xg). After mitochondrial transfer, the fluorescence signal intensity in UC-MSCs was quantified using Image J in a minimum of three independent experiments. All values are mean ± SEM. N=3. *P < 0.05 vs. normal UC-MSCs (0 μg). (B, C) Confocal microscopy images showing intracellular fluorescence of transferred mitochondria (green) in rat L6 muscle cells prelabelled with a red MitoTracker. (B) A representative image prior to mitochondrial transfer; MT (-). (C) A representative image after mitochondrial transfer; MT (+). Red: endogenous mitochondria of L6 cells, green: transferred mitochondria of UC-MSCs, blue: nuclear staining with DAPI, yellow: merged mitochondria. Scale bar, 10 μm. (D, E) Comparison of the transfer efficiency, as determined by the intensity of green fluorescence, following treatment with various amounts of PF-68 for various amounts of time. (D) Representative results showing the uptake efficiency upon co-incubation at 37°C without centrifugation (D) and upon centrifugation for 5 min (E) following treatment of recipient cells with various concentrations of PF-68 (10, 20 and 30 mg/ml) for various amounts of time (0, 1 and 2 h).

Supplementary Figure S3. Analysis of metabolic changes in normal cells after mitochondrial transfer.
Various parameters of normal UC-MSCs were assessed at 48 h after transfer of various amounts of mitochondria (expressed as μg of protein). Changes in intracellular ROS (A) by MitoSOX red (upper) and DCF-DA green (lower) probes, the intracellular ATP content (B) and cell proliferation (C) were analysed and compared. Scale bar, 25 μm. (D) Immunoblot analysis of cytochrome c (Cyto C) in the total cell lysate of UC-MSCs, and cytosolic fraction obtained from UC-MSCs after mitochondrial transfer. (E) Immunoblot analysis using antibodies against Mfn-2 (a mitochondrial fusion marker), DRP-1 (a mitochondrial fission marker), PINK-1 (a mitophagy marker), microtubule-associated protein 1A/1B-light chain 3 (LC3B, an autophagy marker) and β-actin (a cytosolic marker). Representative western blots are shown, along with quantification of the expression levels normalised to that of β-actin. (F) OXPHOS activity. OCRs (pmol/min) were measured in triplicate in three experiments. The OCRs of basal respiration (G), maximal respiration (H), ATP production (I) and spare respiratory capacity (J) are shown. All data represent the mean ± SEM. N=3, *P < 0.05 vs. control (normal UC-MSCs). The grouping of blots were obtained from different parts of the same gel. Full length image of results by Western blot were represented in Fig. S7.

Supplementary Figure S4. Metabolic changes in oligomycin-treated UC-MSCs after delivery of intact mitochondria.
Oligomycin-treated cells were analysed at 48 h after transfer of various amounts of mitochondria (expressed as μg of protein). To induce mitochondrial dysfunction, UC-MSCs were treated with 50 μM oligomycin for 24 h prior to mitochondrial transfer. Changes in cell proliferation (A), the intracellular ATP content (B), the MMP (C) and mitochondrial ROS (D) were analysed and compared under serum-free conditions. (E, F) Immunoblot analysis of AMPK (E) and PGC-1α (F). Representative western blots are shown, along with quantification of the expression levels normalised to that of β-actin. All data represent the mean ± SEM. N=3, *P < 0.05 vs. oligomycin-treated group. The grouping of blots were obtained from different parts of the same gel. Full length image of results by Western blot were represented in Fig. S7.

Supplementary Figure S5. Characterisation of UC-Rho^0 cells.
Analysis was performed after treatment with EtBr for 6 weeks to confirm the induction of UC-Rho^0 cells. (A) UC-MSCs-specific mtDNA (Cord-mtDNA) and human-specific mtDNA (Universial-mtDNA) were analysed by general PCR. Representative gels showing PCR analysis of h-mtDNA (markers in bp) are shown, together with densitometric analysis of the amplified products. (B) Verification of universial-mtDNA deletion

by serial-passaging of UC-Rho^0 cells compared to normal UC-MSCs. (C) The intracellular ATP content of UC-Rho0 cells was measured by a chemiluminescence assay and compared with that of non-treated UC-MSCs. (D) Confocal microscopy images showing the mitochondrial network in UC-MSCs and UC-Rho0 cells. Cells were incubated with 300 nM MitoTracker Red CMXRos for 30 min. Scale bar, 100 μm (E) OXPHOS activity. OCRs (pmol/min) in UC-Rho^0 cells were measured in triplicate in three experiments. The OCRs of basal respiration (F), maximal respiration (G), ATP production (H) and spare respiratory capacity (I) are shown. All data represent the mean ± SEM of three independent experiments. N=3, *P < 0.05 vs. non-treated UC-MSCs.

Supplementary Figure S6. Verification of mitochondrial dysfunction after Dexa treatment.

(A) Dexa (1 μM) treatment depleted the intracellular ATP content of L6 muscle cells in a time-dependent manner. (B) Determination of mROS production following Dexa treatment for 24 h using MitoSOX Red staining. All data represent the mean ± SEM. N=3, *P < 0.05 vs. control.

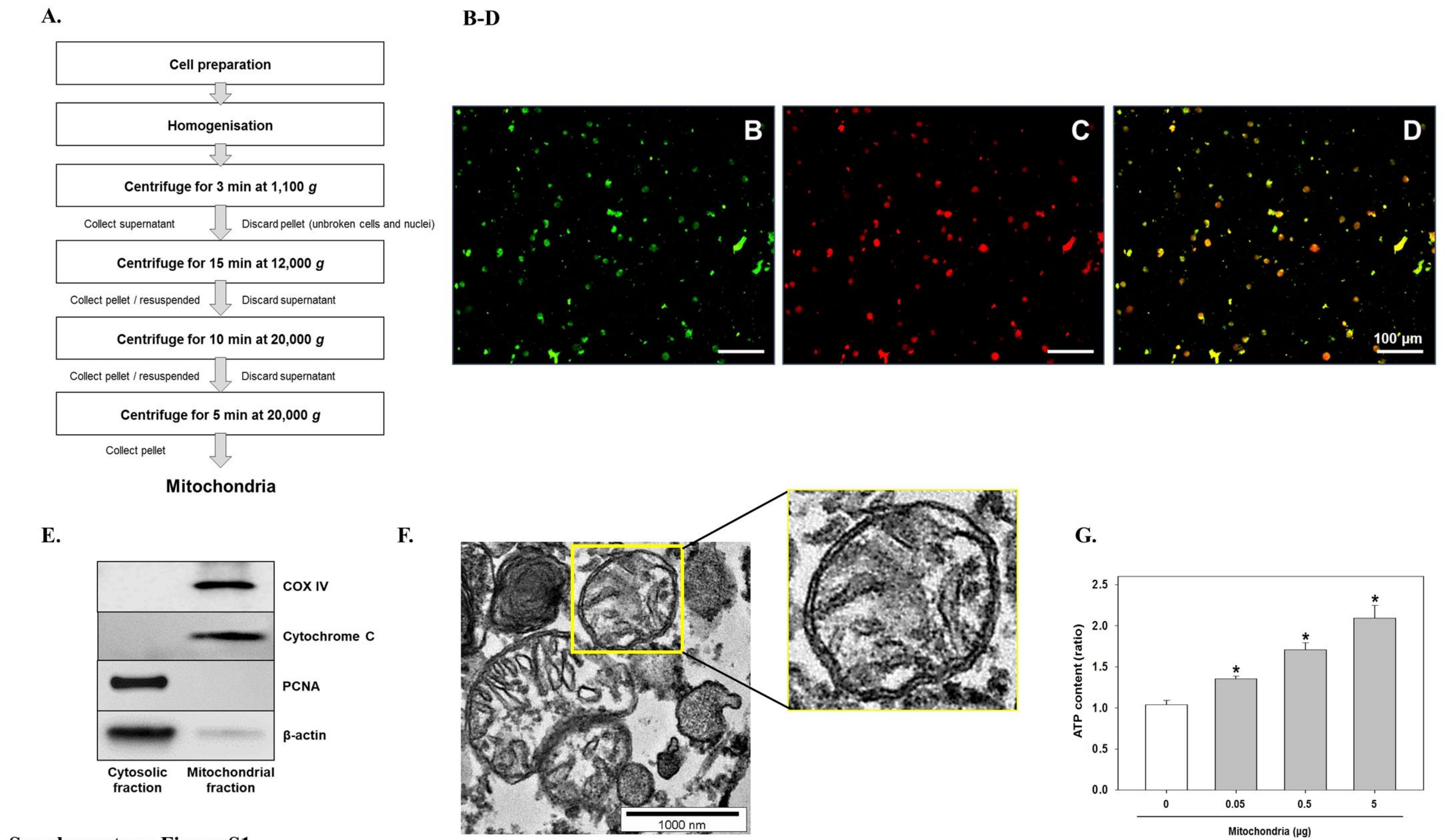

Supplementary Figure S1.

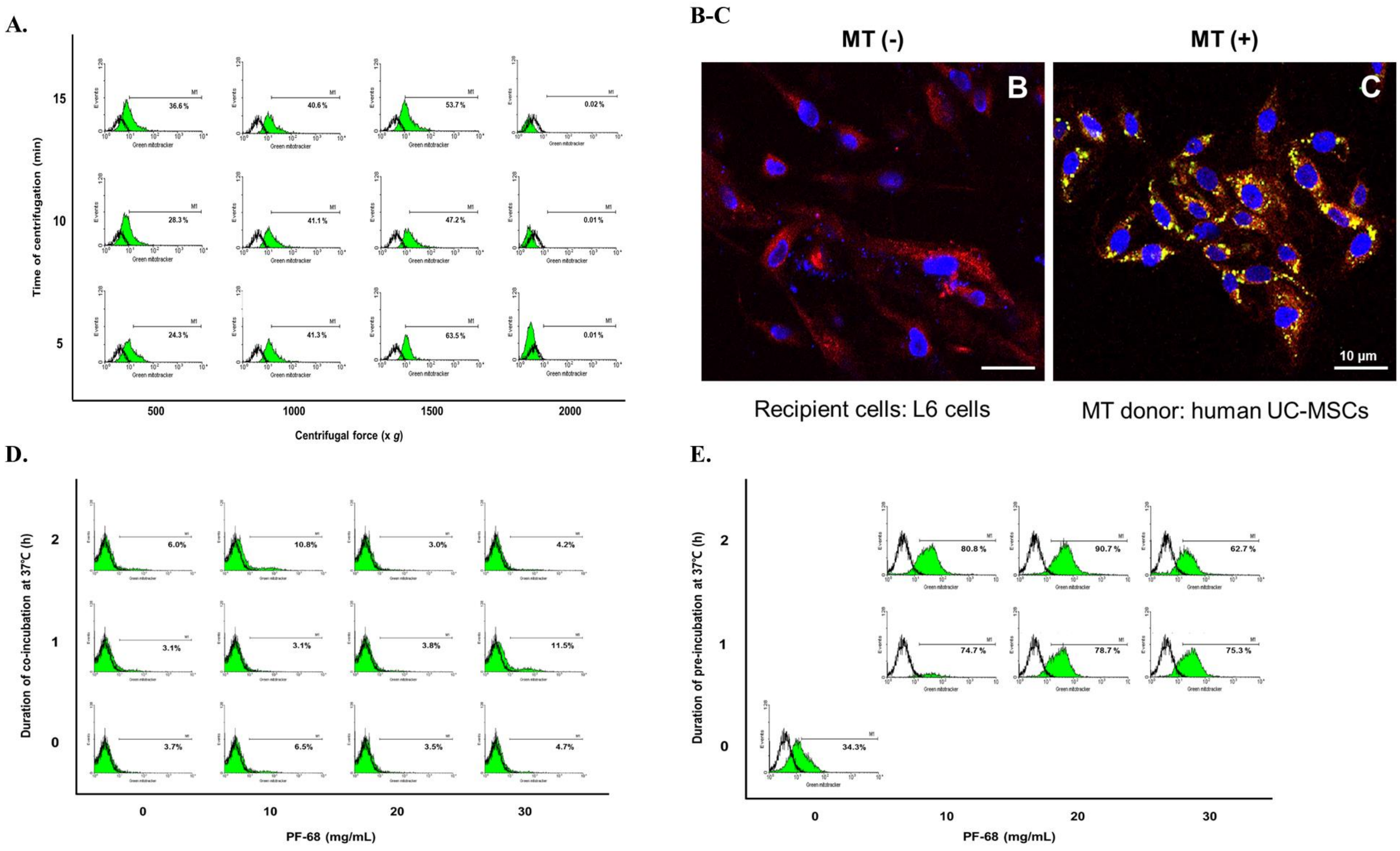

Supplementary Figure S2

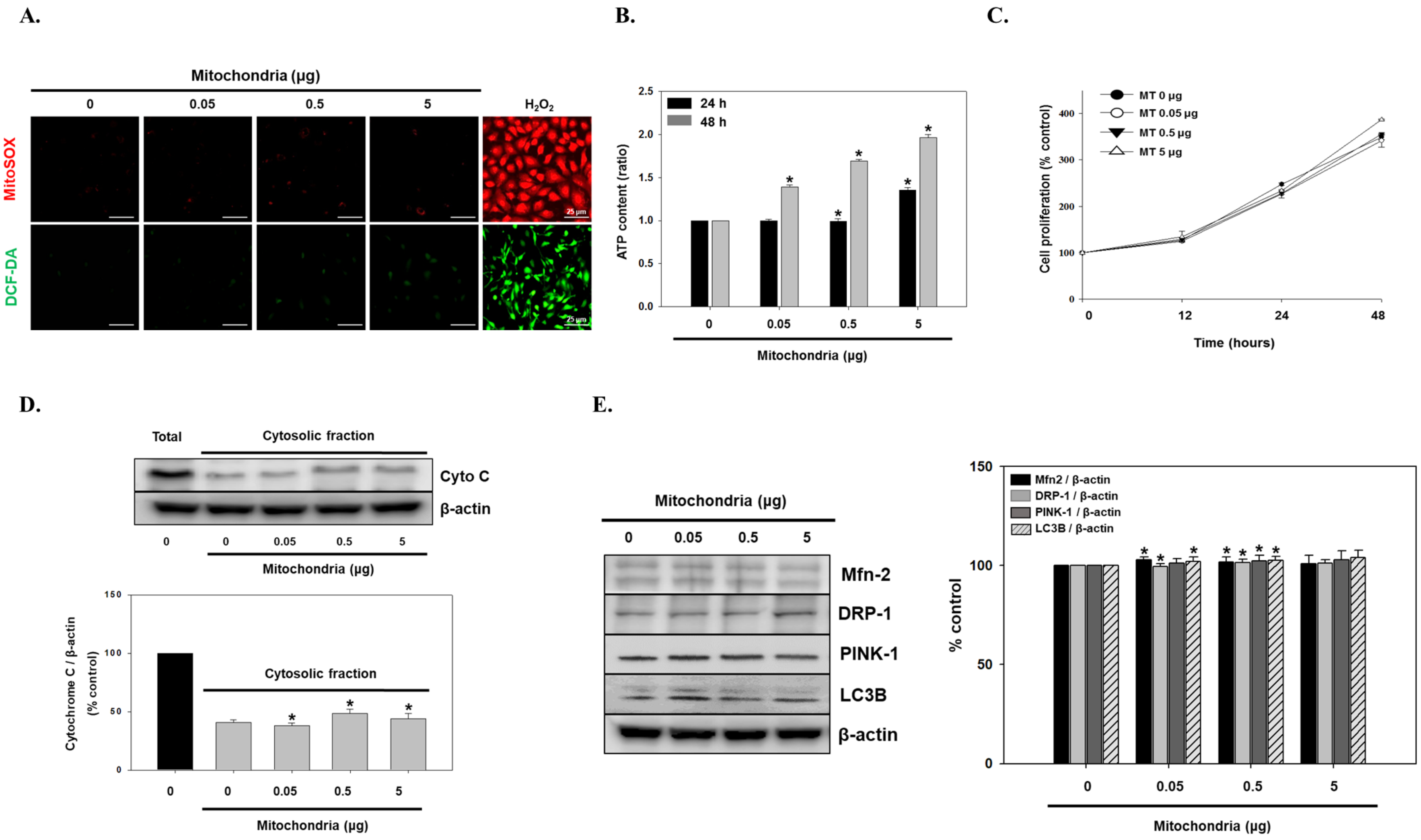

Supplementary Figure S3 (Continued on next page).

F.

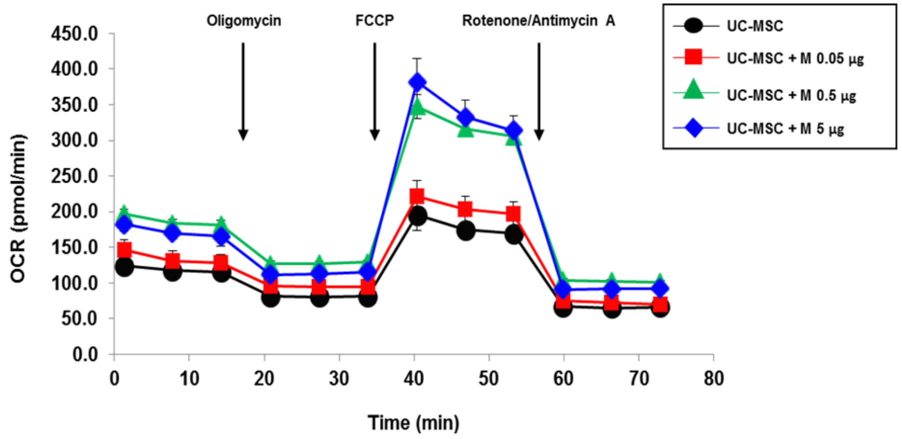

G.

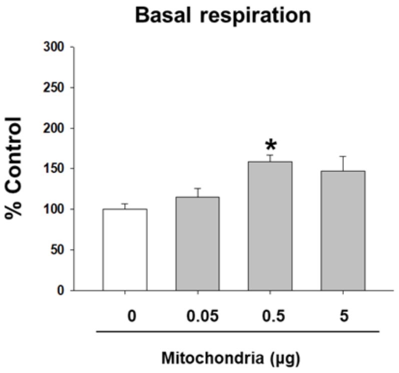

H.

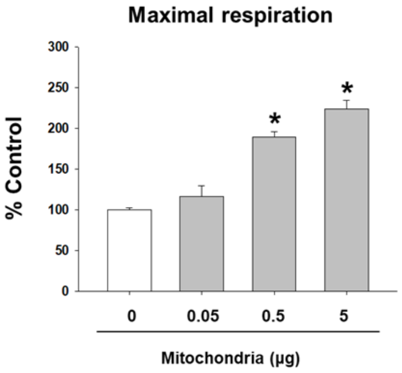

I.

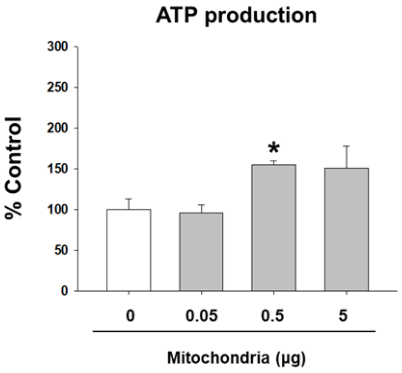

J.

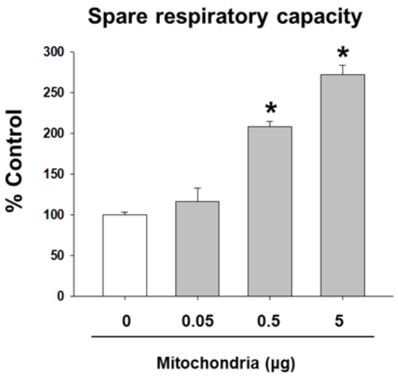

Supplementary Figure S3 (continued).

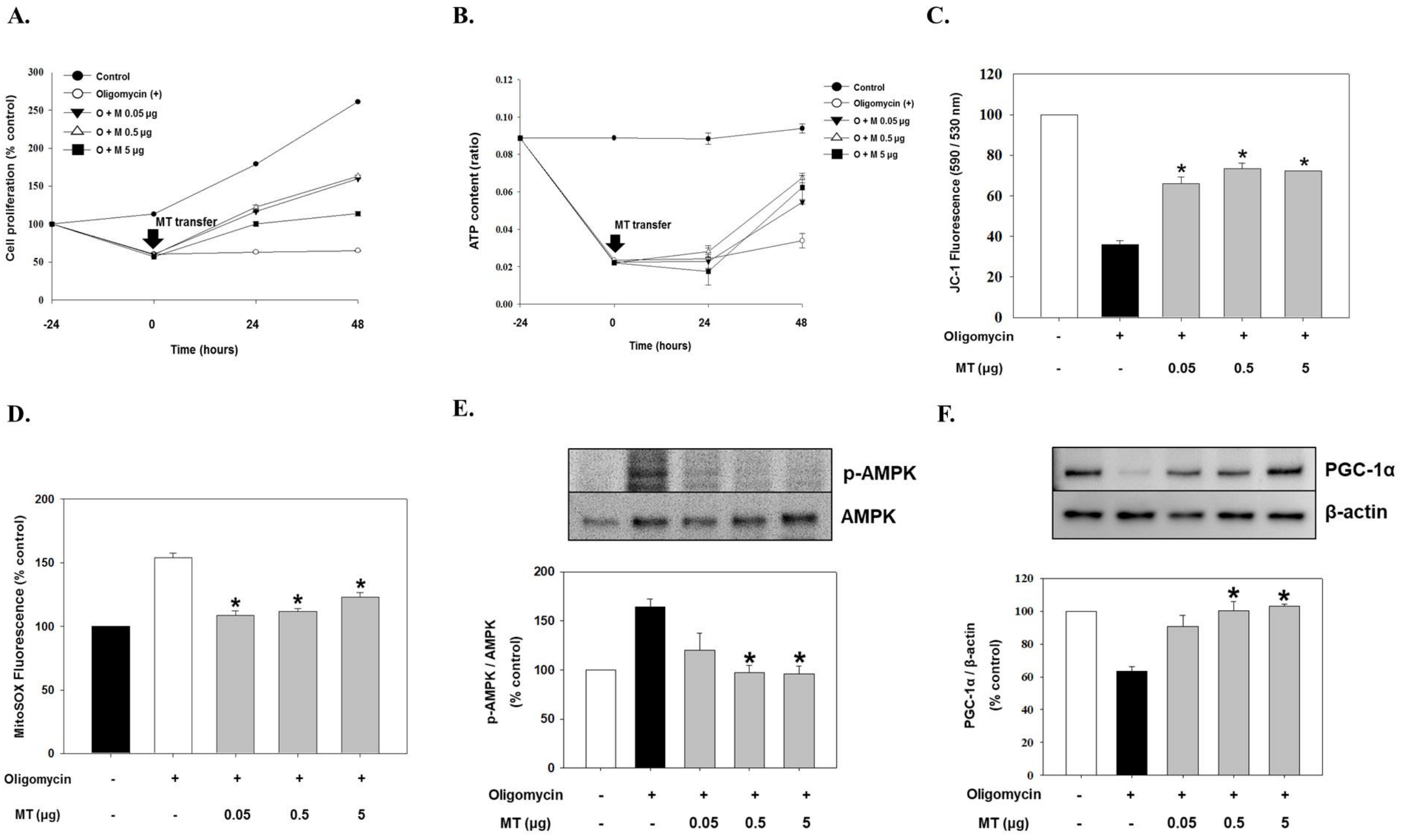

Supplementary Figure S4

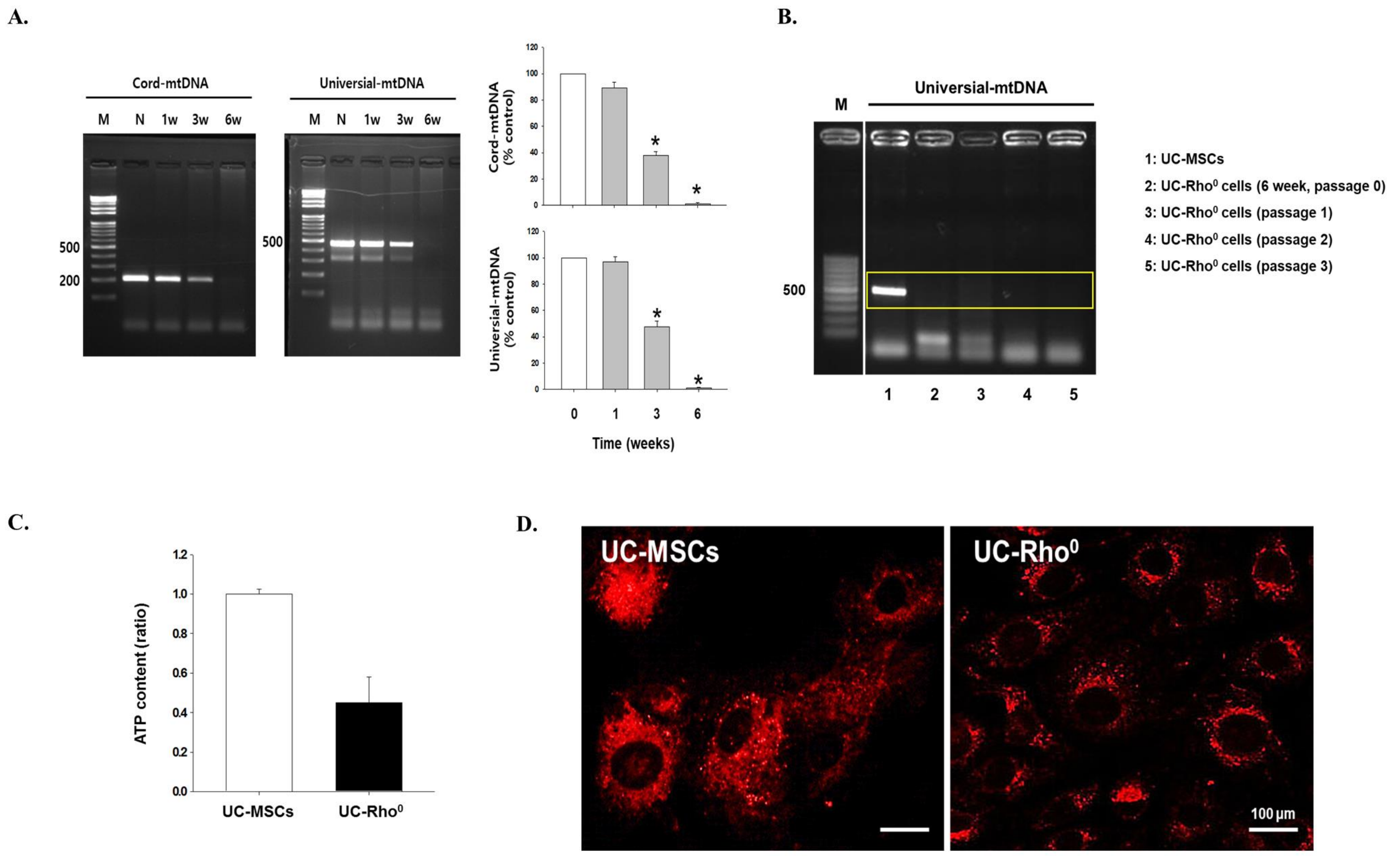

Supplementary Figure S5 (Continued on next page).

E.

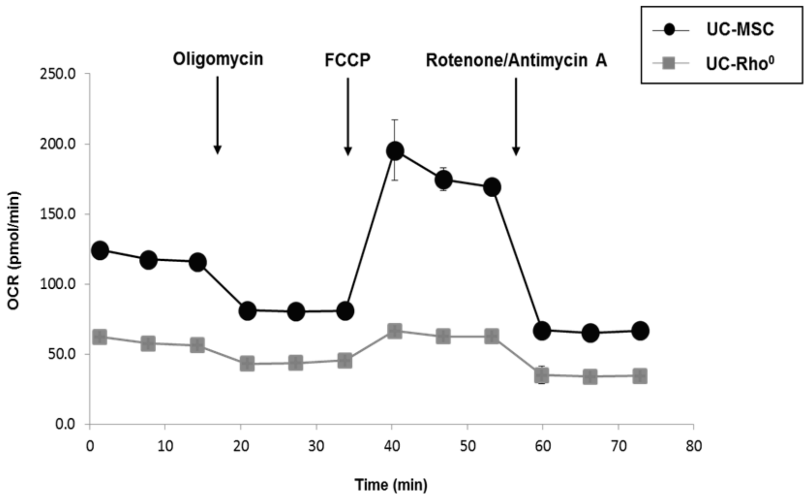

F. G. H. I.

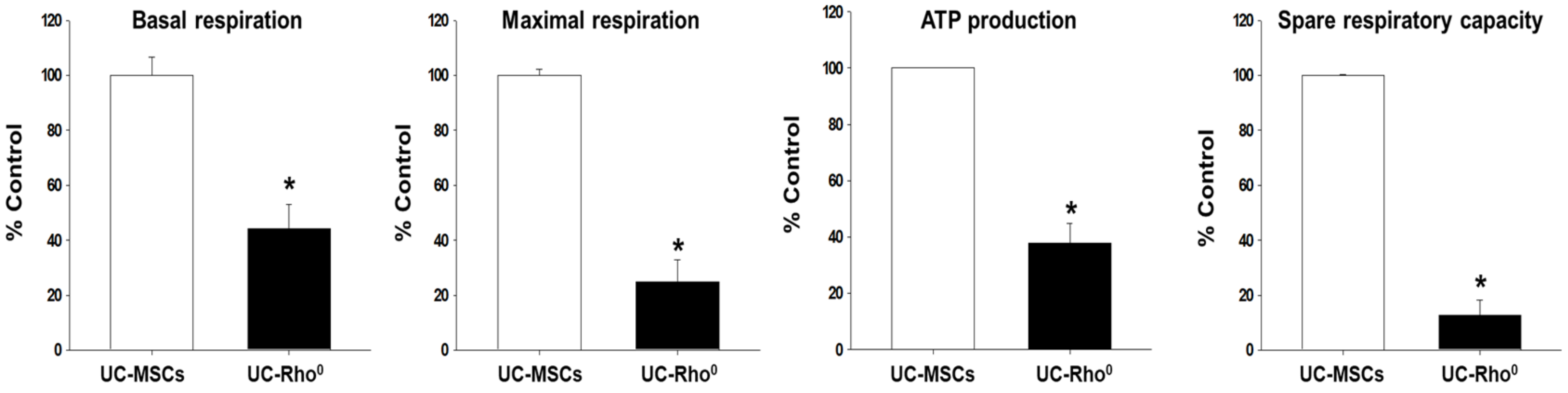

Supplementary Figure S5 (Continued).

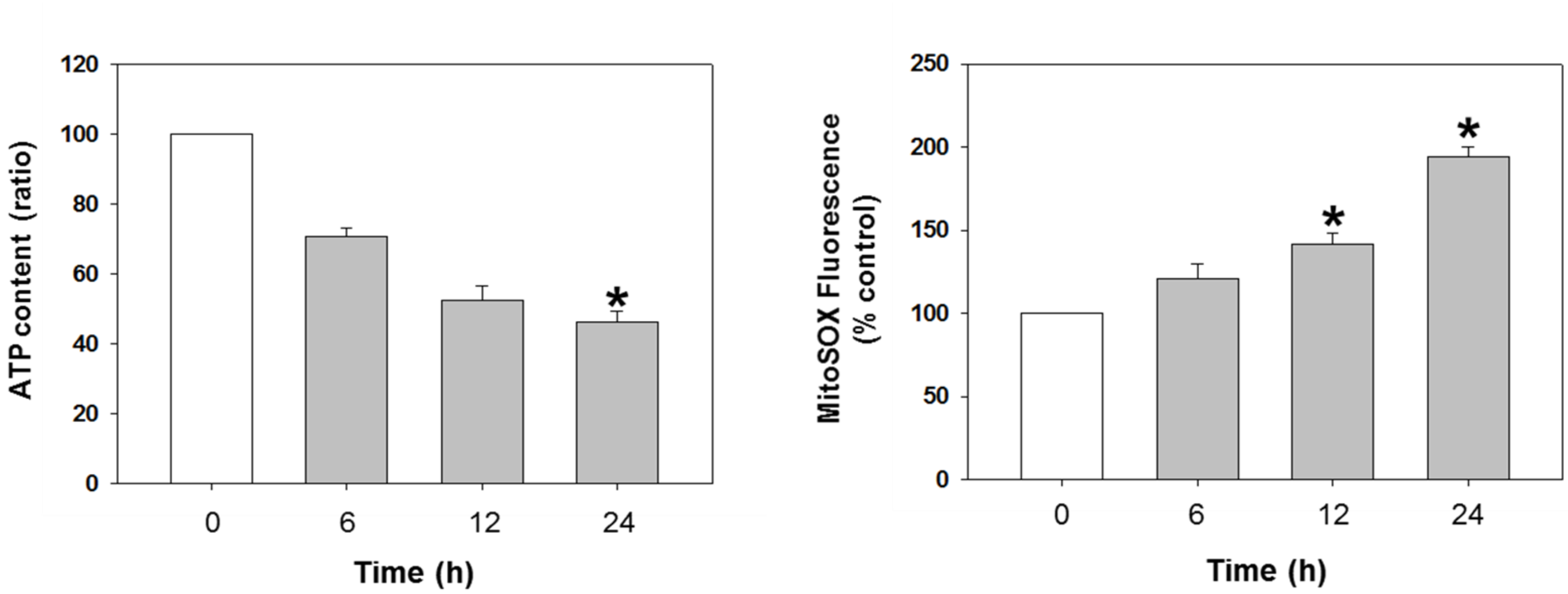

Supplementary Figure S6

Supplementary Figure S7

Full length image of results by Western blot analysis

Fig. 3E

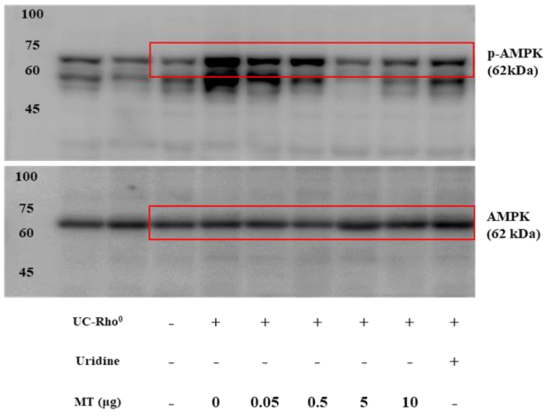

Fig. 3F

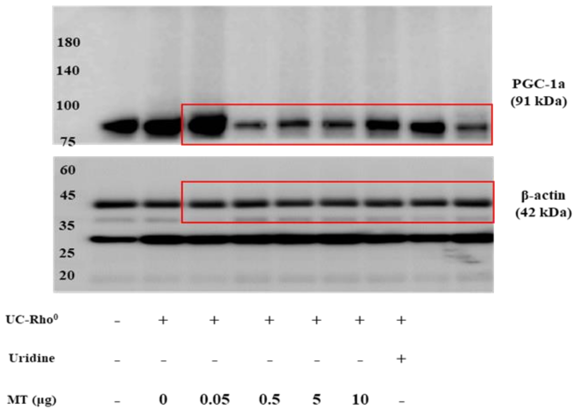

Fig. 4D

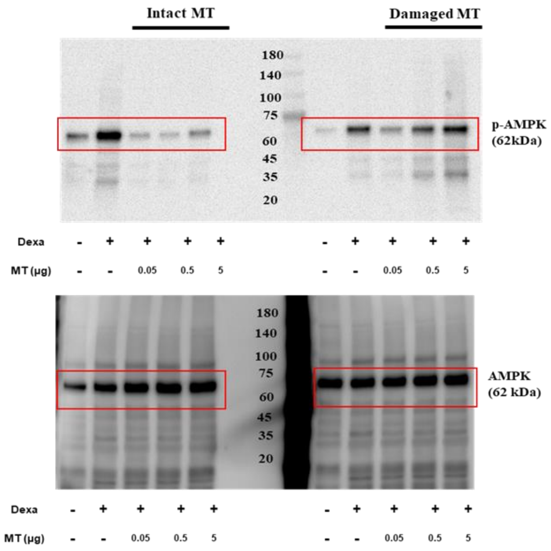

Fig. 4E

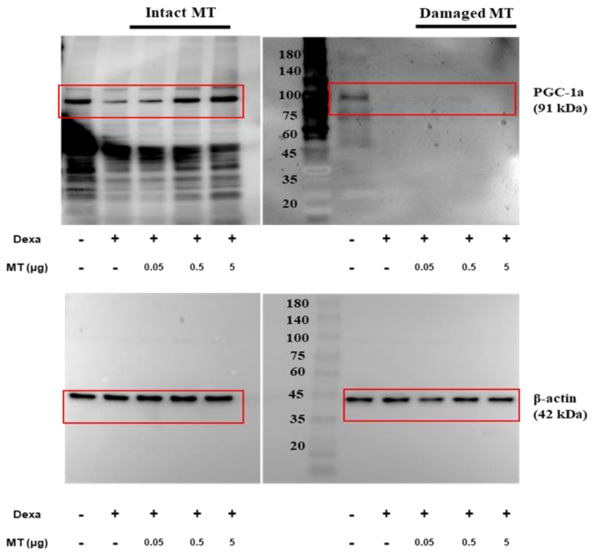

162 **Supplementary Figure S7** **(Continued on next page).**

Fig. 4F

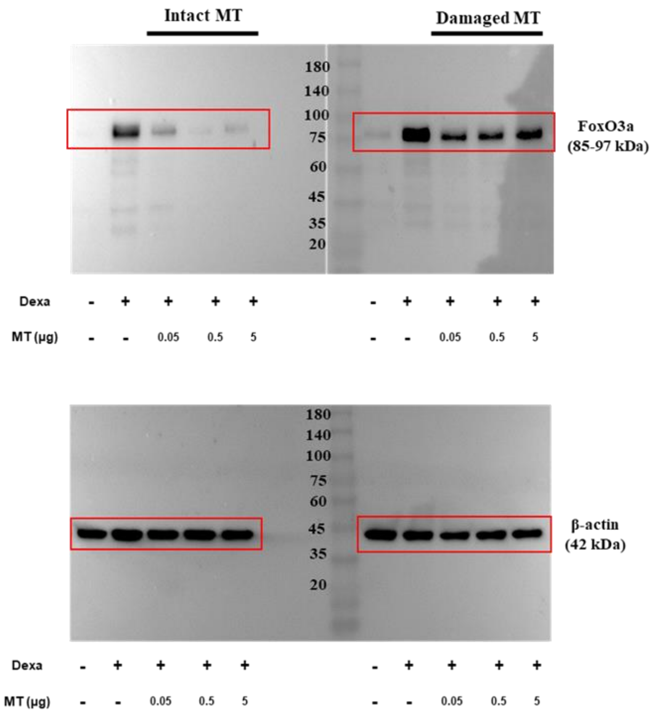

Fig. 4G

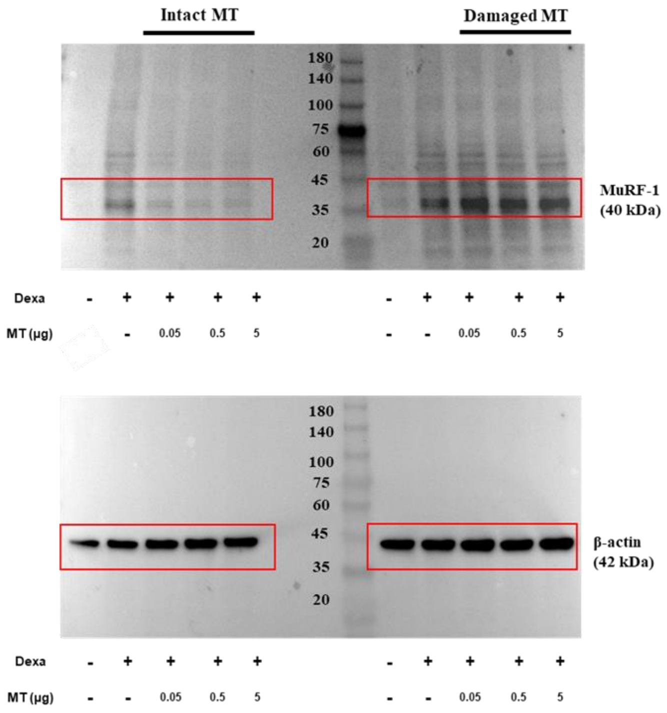

Supplementary Figure S7 (Continued on next page).

Fig. S1E

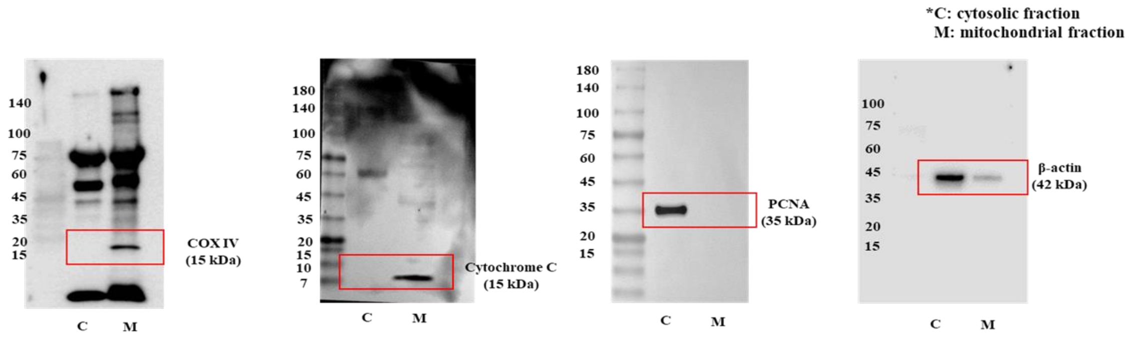

Fig. S3D

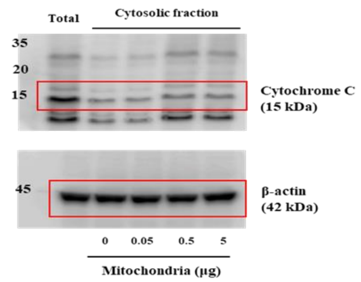

Fig. S3E

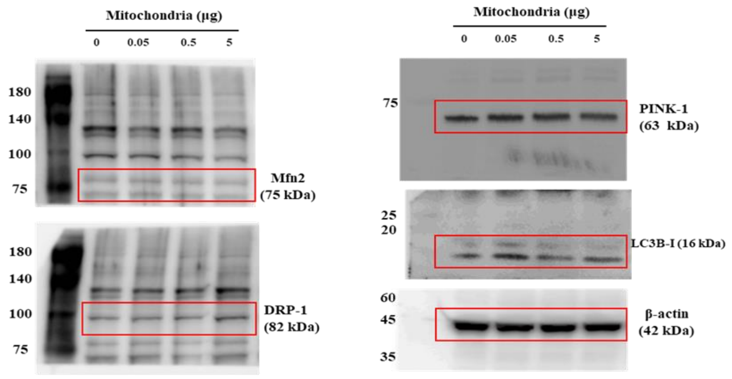

Fig. S4E

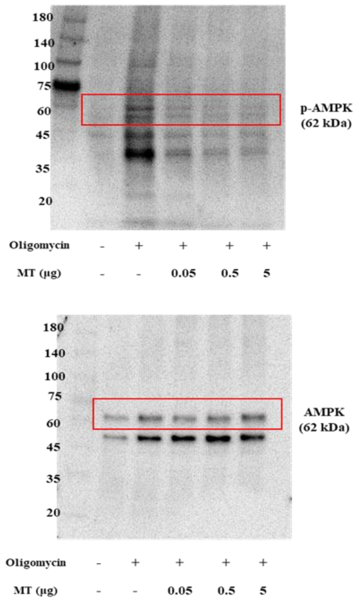

Fig. S4F

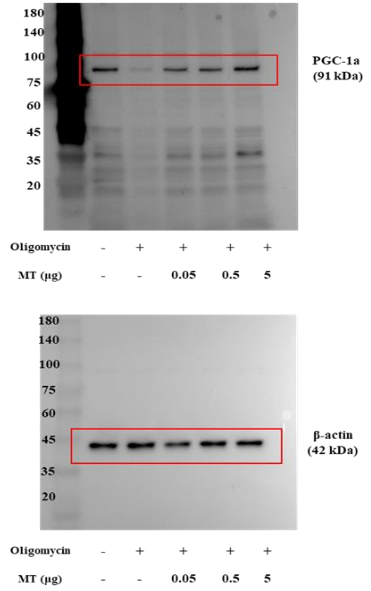

Supplementary Figure S7 (Continued).

Supplementary References

1. Kalyanaraman, B *et al.* Measuring reactive oxygen and nitrogen species with fluorescent probes: challenges and limitations. *Free radic. Biol. Med.* **52**, 1-6, doi:10.1016/j.freeradbiomed.2011.09.030 (2012).
2. Yang, J. *et al.* Prevention of apoptosis by Bcl-2: release of cytochrome c from mitochondria blocked. *Science* **275**, 1129-1132 (1997).
3. Gottlieb, E., Armour, S. M., Harris, M. H. & Thompson, C. B. Mitochondrial membrane potential regulates matrix configuration and cytochrome c release during apoptosis. *Cell death Differ.* **10**, 709-717 (2003).
4. Peng, K. *et al.* Resveratrol Regulates Mitochondrial Biogenesis and Fission/Fusion to Attenuate Rotenone-Induced Neurotoxicity. *Oxid. Med. Cell. Longev.* **2016**, 6705621, doi:10.1155/2016/6705621 (2016).
